# High-performance H_2_/CO_2_ separation from 4-nm-thick oriented Zn_2_(benzimidazole)_4_ films

**DOI:** 10.1126/sciadv.ads6315

**Published:** 2024-12-13

**Authors:** Shuqing Song, Qi Liu, S. Swathilakshmi, Heng-Yu Chi, Zongyao Zhou, Ranadip Goswami, Dmitry Chernyshov, Kumar Varoon Agrawal

**Affiliations:** ^1^ Laboratory of Advanced Separations (LAS), École Polytechnique Fédérale de Lausanne (EPFL), Sion CH-1950, Switzerland.; ^2^ Swiss-Norwegian Beam Lines at European Synchrotron Radiation Facility, Grenoble 38043, France.

## Abstract

High-performance membrane-based H_2_/CO_2_ separation offers a promising way to reduce the energy costs of precombustion capture. Current membranes, often made from two-dimensional laminates like metal-organic frameworks, have limitations due to complex fabrication methods requiring high temperatures, organic solvents, and long synthesis time. These processes often result in poor H_2_/CO_2_ selectivity under pressurized conditions due to defective transport pathways. Here, we introduce a simple, eco-friendly synthesis of ultrathin, intergrown Zn_2_(benzimidazole)_4_ films, as thin as 4 nm. These films are prepared at room temperature using water as the solvent, with a synthesis time of just 10 minutes. By using ultradilute precursor solutions, nucleation is delayed, promoting rapid in-plane growth on a smooth graphene substrate and eliminating defects. These membranes exhibit excellent H_2_ permselectivity under pressurized conditions. The combination of rapid, green synthesis and high-performance separation makes these membranes highly attractive for precombustion applications.

## Teaser

Unit-cell-thick Zn_2_(benzimidazole)_4_ film, prepared in user-friendly manner, yield exceptional performance for H_2_/CO_2_ separation.

## INTRODUCTION

Hydrogen (H_2_), a carbon-free energy carrier, is at the center stage of the renewable energy transition (*1*, *2*). It is produced by reforming fossil fuel followed by high-temperature and low-temperature water-gas shift (WGS) reactions (*3*–*5*). Syngas containing a mixture of H_2_ and carbon dioxide (CO_2_) is then separated into a high-purity H_2_ stream (*6*–*9*). This separation is achieved at a large scale by contacting the H_2_/CO_2_ mixture in a sorbent where CO_2_ is selectively removed (*10*, *11*).

Materials and processes that can improve the energy efficiency of hydrogen purification and reduce the associated carbon capture penalty and related environmental emissions have been intensively investigated (*12*–*15*). High-performance membrane-based separation is highly promising in this context because the membrane process is environment-friendly and does not rely on thermal energy but on electrical energy. High-temperature separation of the H_2_/CO_2_ mixture will allow the integration of the WGS reaction with the separation process, increasing the overall process efficiency (*16*–*18*).

Membrane-based H_2_/CO_2_ separation relies on a selective diffusional pathway of H_2_. Dense palladium membranes yield a high H_2_ permeance [flux normalized by the transmembrane partial pressure difference $\left(∆P_{p}\right)$] and a high selectivity via chemisorption and diffusion of H in interstitial lattice sites (*19*–*22*). However, they face challenges from a high material cost and instability in the presence of hydrocarbon, CO, and H_2_S (*23*). Separation using polymeric membranes benefits from the high processibility of polymers (*24*). However, the state-of-the-art polybenzimidazole-based membranes yield modest H_2_ permeance and H_2_/CO_2_ selectivity (*25*, *26*). This is mainly because the kinetic diameters of H_2_ and CO_2_ (2.89 and 3.30 Å, respectively) are similar, making this separation challenging. CO_2_-selective polymeric membranes are emerging as a promising alternative; however, their performance is optimal at low temperatures (*27*, *28*).

Nanoporous materials are highly promising by advancing the prospects of separations, thanks to the possibility of rapid permselective transport from the intrinsic pores in this class of materials. Membranes based on zeolites (*29*–*34*), metal-organic frameworks (MOFs) (*35*–*40*), and exfoliated two-dimensional (2D) layers [graphene oxide (*41*–*46*), MXenes (*47*–*49*), and covalent-organic frameworks (*50*–*53*)] have shown promising H_2_/CO_2_ separation performances. However, most studies use zero ∆P (total pressure difference across feed and permeate channels) for performance evaluation (*54*–*57*), where a notable selectivity decline is observed upon pressurization, either from the limited stability of transport channels or from the presence of pinhole defects in the selective layer (*58*, *59*). The latter results in a dominant viscous transport, which leads to the loss of selectivity.

A promising approach to preparing a high-quality membrane is to develop porous 2D films with an infinite aspect ratio. This way, challenges from pinhole defects and stacking will be eliminated. Recently, an MOF composed of a zeolitic imidazolate framework (ZIF) was reported as a 2D film with an infinite aspect ratio (*60*). 2DZIF structure consists of a six-membered ring (6-MR) forming a pore aperture that successfully separates H_2_ from N_2_. However, the 6-MR pore in 2DZIF is too large for H_2_/CO_2_ separation. As a result, H_2_/CO_2_ separation could not be achieved.

**Fig. 1.**
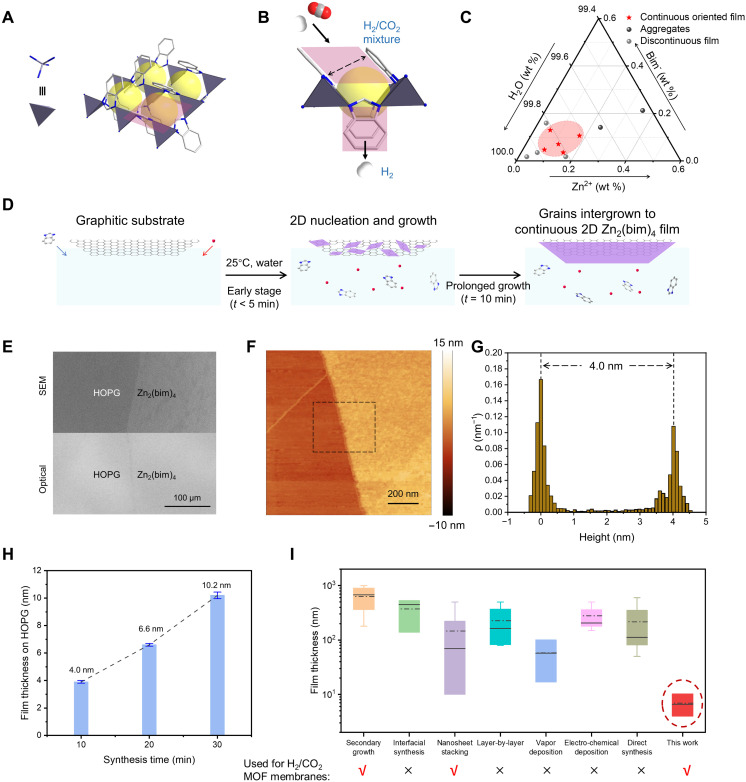
Schematic of Zn_2_(bim)_4_ and the MOF film forming process. (**A**) Structure of Zn_2_(bim)_4_. Zn, C, and N are represented by the purple, white, and blue balls. The 4-MR aperture is highlighted in purple. (**B**) Schematic illustration of the 4-MR for molecular sieving. (**C**) The precursor concentration phase diagram reveals regions where high-quality films could be formed (also see fig. S1). (**D**) Schematic of Zn_2_(bim)_4_ film synthesis. (**E**) Scanning electron (top) and optical (bottom) microscopy images of the Zn_2_(bim)_4_ film. AFM (**F**), and the corresponding height distribution profile (**G**) of a Zn_2_(bim)_4_ film on HOPG, where ρ on Y-axis denotes frequency for AFM height. Height distribution is acquired from the area in the black box in (F). (**H**) Zn_2_(bim)_4_ films on HOPG with varying thicknesses as a function of the synthesis time. (**I**) Comparison of the thicknesses of the reported MOF membranes fabricated via different methods (see table S1). Synthesis techniques yielding H_2_/CO_2_ selective MOFs have been highlighted.

A promising H_2_/CO_2_ separation material is Zn_2_(benzimidazole)_4_ [Zn_2_(bim)_4_]. It consists of a small pore aperture formed by a four-membered ring (4-MR) along the *c* axis with a crystallographic dimension of 2.1 Å (*61*). The aperture is expected to expand to accommodate guest molecules, thanks to the linker flexibility (Fig. 1B) (*62*–*64*). The first example of 2DMOF membrane used Zn_2_(bim)_4_ as the selective layer where membranes were prepared by stacking exfoliated Zn_2_(bim)_4_ nanosheets that had an aspect ratio of ~500 (*35*, *65*). However, these membranes could not be pressurized, likely due to changes in stacked nanosheet structure resulting from the limited aspect ratio of the exfoliated nanosheets. Apart from the pressurization issue, the synthesis of the Zn_2_(bim)_4_ membrane follows a lengthy multistep approach. Its precursor (ZIF-7, phase 1) is synthesized in an organic solvent at high temperatures (*66*). This is followed by a hydrothermal process that converts ZIF-7 into the lamellar Zn_2_(bim)_4_ (*67*). Nanosheets are then obtained by exfoliating the lamellar precursor (*35*). A coating suspension is prepared, and membranes are prepared by stacking nanosheets.

Herein, we report a synthesis route for the oriented 2D Zn_2_(bim)_4_ film hosting an infinite aspect ratio. The synthesis is user-friendly and carried out at room temperature in an aqueous precursor solution in a few minutes. An ultrathin, oriented, pinhole-free film with a thickness of 4 nm could be achieved. 4-MR apertures in 2D Zn_2_(bim)_4_ films are perpendicular to the transport direction. The resulting membranes demonstrate superior performance compared to state-of-the-art membranes, particularly under high-temperature, pressurized feed conditions.

## RESULTS

### Synthesis of the 2D Zn_2_(bim)_4_ film

Zn_2_(bim)_4_ is a zinc-based monoclinic MOF composed of zinc as the metal node and bim as the linker (Fig. 1A) (*61*, *67*). The first hurdle in achieving oriented, in-plane growth is homogeneous nucleation where a nucleus formed in the bulk solution can attach onto the growing film, with random orientation, disrupting the oriented growth. The second hurdle is the substrate, where the roughness of the substrate can hamper the in-plane growth, especially for synthesizing unit cell–thick films. These hurdles were overcome using ultradilute growth precursor solution and atomic-smooth substrate. The former delays homogeneous nucleation, whereas the latter provides smoothness to propagate the in-plane growth of the oriented film. With this strategy, the synthesis of the unit cell–thick Zn_2_(bim)_4_ film could be achieved for the first time.

For effective film growth, precursor composition was screened. This involved a study of the morphology of the film as a function of precursor concentration and the metal/linker ratio (note S1). The film was deposited on a substrate by immersing the substrate in the precursor solution. The optimal Zn^2+^ and bim^−^ concentrations were between 4.7 to 9.4 mM and 4.5 to 9.0 mM, respectively, corresponding to a metal/linker ratio ranging from 0.5 to 2.1 (Fig. 1C and fig. S1). Within this composition window, a uniform and continuous layer was formed on various graphitic substrates (Fig. 1D), including highly oriented pyrolytic graphite (HOPG, fig. S2), chemical vapor deposition (CVD)–derived single-layer graphene resting on Cu foil (fig. S3), and CVD graphene transferred on a Si/SiO_2_ wafer (fig. S4).

The Zn_2_(bim)_4_ film, prepared on HOPG with a reaction time of 10 min, was analyzed using optical and scanning electron microscopy (SEM, Fig. 1E). A distinct and uniform contrast could be observed between the film and the substrate (fig. S5). The uniformity of the contrast suggests that the film is smooth, continuous, and macroscopically uniform. Further examination by atomic force microscopy (AFM) at the edge of the film revealed a thickness of ~4 nm (Fig. 1, F and G). This thickness corresponds to two unit cells of Zn_2_(bim)_4_ along the *c* out-of-plane direction (fig. S6). By extending the growth time to 20 and 30 min, films with thicknesses of ~6.6 and ~10.2 nm, respectively, could be synthesized (Fig. 1H and fig. S7). Increasing growth time beyond 30 min resulted in the deposition of randomly oriented crystals without further increase in the film thickness (fig. S8). This indicates the onset of homogeneous nucleation and its competition with the growth of the film at this stage.

Compared with reported polycrystalline MOF films synthesized via emerging deposition techniques (Fig. 1I and table S1), the Zn_2_(bim)_4_ film can be uniquely synthesized with a thickness down to just two unit cells. Among the array of MOF membrane synthesis methods for H_2_/CO_2_ separation, this approach is the only method that yields film thickness below 10 nm. This enhances the potential for achieving high performance in H_2_/CO_2_ separation. In conclusion, 2D Zn_2_(bim)_4_ film with an aspect ratio of infinity was successfully synthesized. Pinhole-free films with thicknesses as small as two unit cells could be prepared, overcoming challenges from the tendency of nonoriented film growth.

### Structure of the 2D Zn_2_(bim)_4_ film

**Fig. 2.**
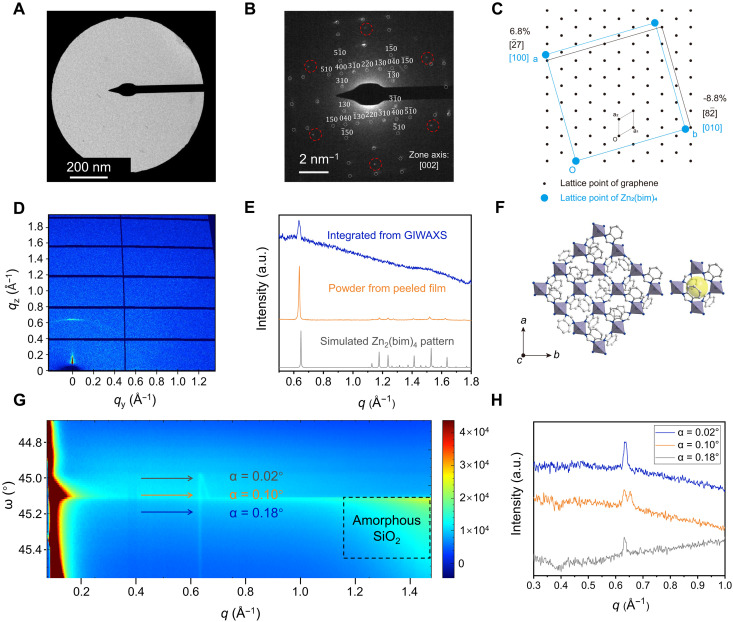
Structure solution of the 2D Zn_2_(bim)_4_ film. (**A**) Bright-field TEM image of the 2D Zn_2_(bim)_4_ film grown on suspended graphene, and (**B**) the corresponding SAED pattern. The patterns from graphene are identified with red circles, and those from Zn_2_(bim)_4_ are identified with white circles. (**C**) Registry between one unit cell of Zn_2_(bim)_4_ and a supercell of graphene based on SAED data. (**D**) 2D GIWAXS intensity images (λ = 1.04157 Å) of Zn_2_(bim)_4_ film (~12 nm) prepared on graphene/SiO_2_/Si, with an incidence angle of 0.02°. (**E**) XRD data from GIWAXS (top, blue), powder from the peeled film (middle, orange), and simulated Zn_2_(bim)_4_ pattern (bottom, gray). (**F**) The c-oriented Zn_2_(bim)_4_ structure and visualization of 4-MR (right); Zn, C, and N are represented with the purple, white, and blue balls. (**G**) GIWAXS patterns of the Zn_2_(bim)_4_ film at various incidence angles (α). The incidence angle was adjusted by altering the rotation angle (ω) and determined by calculating the angle difference between the sample surface and the beam (for details, see fig. S14). The area circled by the black dashed line shows the wide and nonoriented peaks derived from amorphous SiO_2_ on the substrate (fig. S17). (**H**) The intensity of the 002 peak of Zn_2_(bim)_4_ film at three incidence angles at 0.02° (top, blue), 0.10° (middle, orange), and 0.18° (bottom, gray).

To understand the structure of the 2D Zn_2_(bim)_4_ film, it was deposited on suspended graphene resting on a holey transmission electron microscopy (TEM) grid. Bright-field TEM image of the film revealed that the film is homogeneous and free from deposits of macroscopic Zn_2_(bim)_4_ crystals (Fig. 2A). Selected area electron diffraction (SAED) pattern from the film, obtained from a micrometer-sized area, yielded sharp diffraction spots. These spots could be isolated in two sets (Fig. 2B). The first set (Fig. 2B, red circles) displays a sixfold symmetry, representing a single grain of graphene. In contrast, the other set (white circles) exhibits a twofold symmetry, representing a single grain of 2D Zn_2_(bim)_4_ in a micrometer area. This indicates that the grain size of 2D Zn_2_(bim)_4_ was at least 1 μm. Indexing of SAED patterns confirms [002] zone axis concerning the 2D Zn_2_(bim)_4_ film (Fig. 2B and note S2). The diffraction pattern assigns *a* and *b* lattice parameters as 1.62 and 1.60 nm, respectively, in close agreement with the simulated lattice parameter (*a* = 1.61 nm, *b* = 1.61 nm, table S2). This SAED pattern was obtained from every location in the entire TEM grid, indicating that the crystal growth was uniform. The lattice parameters of Zn_2_(bim)_4_ match with those of a graphene supercell (Fig. 2C, fig. S9, and note S3), indicating a registry between graphene and Zn_2_(bim)_4_ film.

Synchrotron grazing-incidence x-ray diffraction (GIXRD) on a ~12-nm-thick 2D Zn_2_(bim)_4_ film, prepared on graphene resting on a Si/SiO_2_ wafer (fig. S10), yielded a sharp diffraction peak in the out-of-plane direction, indicating a strong preferential orientation (Fig. 2D). Integration of the GIWAXS pattern yielded a clear peak at *q* = 0.633 Å^−1^ (Fig. 2E) consistent with the 002 reflection of Zn_2_(bim)_4_. This confirms that the film was macroscopically uniform. We carried out azimuthal integration of the GIWAXS pattern along the 002 peak, as illustrated in fig. S11A. The resulting plot (fig. S11B) reveals that the peak intensity 002 is most pronounced at a precise azimuth angle of 90°. This observation suggests a distinct preference for out-of-plane orientation.

To characterize the crystal structure of the film material, the film was peeled off from the substrate and crushed to a powder. Analysis of this powder by x-ray diffraction (XRD) shows other peaks expected with the structure of Zn_2_(bim)_4_ (Fig. 2E). Rietveld refinement of the data confirmed that the as-prepared film is indeed Zn_2_(bim)_4_ (fig. S12). Scherrer equation analysis indicates that the as-prepared Zn_2_(bim)_4_ film is composed of large grains (>500 nm) consistent with the SAED data (fig. S13). On the basis of this and the SAED data, it can be confirmed that the 2D Zn_2_(bim)_4_ film is *c*-oriented with its 4-MR pore aperture parallel to the substrate wherein the direction of molecular transport (Fig. 2F) is attractive for H_2_/CO_2_ separation.

To gain further insights into the crystallinity of the 2D Zn_2_(bim)_4_ film, the GIWAXS configuration was optimized by changing the rotation angle of the sample (ω) with the incident angle (α) from 0° to 0.3° (Fig. 2G and fig. S14). A double peak effect becomes pronounced for α in the range of 0.06° to 0.14° (Fig. 2, G and H, and fig. S15). Figure S16 depicts the beam trajectories responsible for the observed dual peak phenomenon. This occurs due to deviations in the paths of the beam as it interacts with the film: either first scattered, then reflected from the substrate, or vice versa (*68*, *69*). At α of 0.14°, the dual peaks converge, shifting toward higher *q* values as α increases (fig. S15). This optical phenomenon provides further support for the assertion that the Zn_2_(bim)_4_ film exhibits uniform and homogeneous epitaxial deposition and high crystallinity when deposited on graphitic substrates. Meanwhile, at an incidence angle exceeding 0.16°, a robust and broad peak emerges within a *q* value range of approximately 1.0 to 2.5 Å^−1^, lacking any discernible orientation (Fig. 2G and fig. S15). This peak corresponds to the amorphous SiO_2_ layer on the Si wafer (fig. S17).

X-ray photoelectron spectroscopy (XPS) of 2D Zn_2_(bim)_4_ was carried out to understand the coordination environment in the film (fig. S18). The high-resolution spectrum of Zn 2*p* has two prominent peaks corresponding to Zn 2*p*_1/2_ (1023.7 eV) and Zn 2*p*_3/2_ (1046.7 eV). Each main peak can be further resolved into two subpeaks representing fully coordinated (1023.7 and 1046.7 eV) and unsaturated zinc (1025.1 and 1048.52 eV) (*70*). This distinction arises when either the crystal terminates with zinc at its edge or grain boundary or defects are generated due to the absence of the bim linker in the ZnN_4_ tetrahedra (*71*). In summary, analysis via TEM, SAED, XRD, and XPS confirmed that the 2D Zn_2_(bim)_4_ film is crystalline, uniform, smooth, and *c*-out-of-plane oriented with crystal structure consistent with the theoretical structure of Zn_2_(bim)_4_.

### Early stages of growth of the Zn_2_(bim)_4_ film

**Fig. 3.**
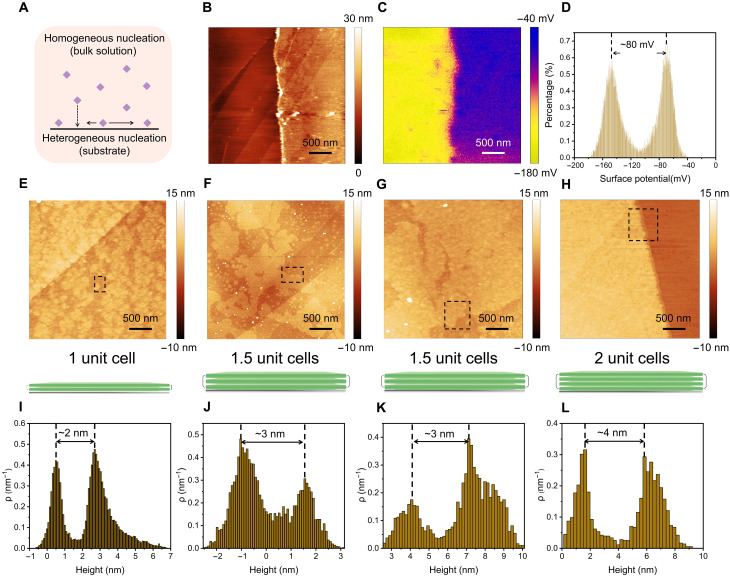
In-plane epitaxial growth of the Zn_2_(bim)_4_ film on HOPG. (**A**) Illustration of the in-plane growth process on the substrate in the ultradilute precursor solution. (**B**) AFM and (**C**) KPFM images of the 10-min growth Zn_2_(bim)_4_ film on HOPG, and (**D**) the corresponding surface potential profile. AFM images of Zn_2_(bim)_4_ film on HOPG with a growth time of (**E**) 4 min, (**F**) 6 min, (**G**) 8 min, and (**H**) 10 min, respectively. (**I** to **L**) The corresponding height profile of the black dashed circled area in (E) to (H), respectively.

Immersing a substrate in the precursor solution initiates heterogeneous nucleation and growth on the substrate as well as homogeneous nucleation of crystals in the solution (Fig. 3A). By using an ultradilute precursor solution (Fig. 1C), one can eliminate or delay homogeneous nucleation. Otherwise, the nuclei from the bulk can deposit on the substrate and promote out-of-plane nonoriented growth. To understand film growth and the possibility of the influence of bulk crystals deposited from the solution, film morphology was analyzed at the early stage of growth (2 to 10 min). Kelvin probe force microscopy (KPFM) near the edge of the 2D Zn_2_(bim)_4_ film on HOPG revealed a distinct surface potential difference (80 mV) between the film and the substrate (Fig. 3, B to D). This confirmed the presence of the 2D Zn_2_(bim)_4_ film on the substrate and paved the way to carry out AFM analysis on the early stages of film growth.

At 2 min of growth, 100- to 200-nm-sized grains appear on HOPG (figs. S19A and S20) with a thickness of ~2 nm, consistent with a unit-cell-thick Zn_2_(bim)_4_ grain oriented along the *c*-out-of-plane direction. When the growth time was extended to 4 min, the grains merged into larger islands on the substrate (Fig. 3E and fig. S19B) with thickness remaining ~2 nm (Fig. 3I). After 6 min, the lateral size of these islands enlarged (Fig. 3F and fig. S19C) with the thickness increasing to ~3 nm, suggesting growth to a 1.5 unit cell thickness (Fig. 3J). The 1.5-unit-cell-thick Zn_2_(bim)_4_ is essentially three layers of the MOF where two layers constitute a single unit cell. The grains continued to enlarge at 8 min (Fig. 3G and fig. S19D), leading to a film that covered more than 80% of the substrate. At this point, the film thickness was maintained at ~3 nm (Fig. 3K). The islands intergrew to yield a continuous film after 10 min of growth (Fig. 3H) while the thickness increased to ~4 nm, corresponding to two unit cells (Fig. 3L). This demonstrates predominant in-plane grain growth (length scale ~1 μm), which was more than 100-fold faster than the growth along the out-of-plane direction (length scale ~4 nm). Over time, the in-plane growth led to a continuous film, while a slow out-of-plane growth resulted in extremely thin film. Analysis of grains, grown for 2 to 8 min, by SAED indicated that the grains are indeed crystalline (fig. S21). The analysis by AFM and SAED confirms that 2D Zn_2_(bim)_4_ film preferentially grow along the in-plane direction with film growth progressing from a submonolayer film composed of discrete grains to an intergrown film covering the entire substrate.

### H_2_/CO_2_ separation performance of the 2D Zn_2_(bim)_4_ film

To investigate the separation performance of the 2D Zn_2_(bim)_4_ film, the film was deposited on nanoporous graphene (NG), where the latter was prepared by O_2_-plasma treatment to incorporate large pores [2.4 ± 1.5 nm (*72*, *73*)] in graphene. These graphene pores are notably larger than H_2_ and CO_2_, eliminating the possibility of molecular sieving. NG was mechanically reinforced with ~250-nm-thick high-flux polymeric film (fig. S22). Hereafter, NG/polymeric film is referred to as the support film. This support film yielded a high H_2_ permeance [>16,000 gas permeation units (GPU), 1 GPU = 3.35 × 10^−10^ mol m^−2^ s^−1^ Pa^−1^]. As expected, the support film was not selective for H_2_/CO_2_ separation (selectivity <1, fig. S23).

A 4-nm-thick 2D Zn_2_(bim)_4_ film was grown on the support film, forming the selective layer of the membrane. The 2D Zn_2_(bim)_4_ film was then collected on a macroporous metal foil support (fig. S24). In this way, the 2D Zn_2_(bim)_4_ film was sandwiched between the metal foil and the support film (fig. S25) with stacking order of metal foil/2D Zn_2_(bim)_4_/support film.

**Fig. 4.**
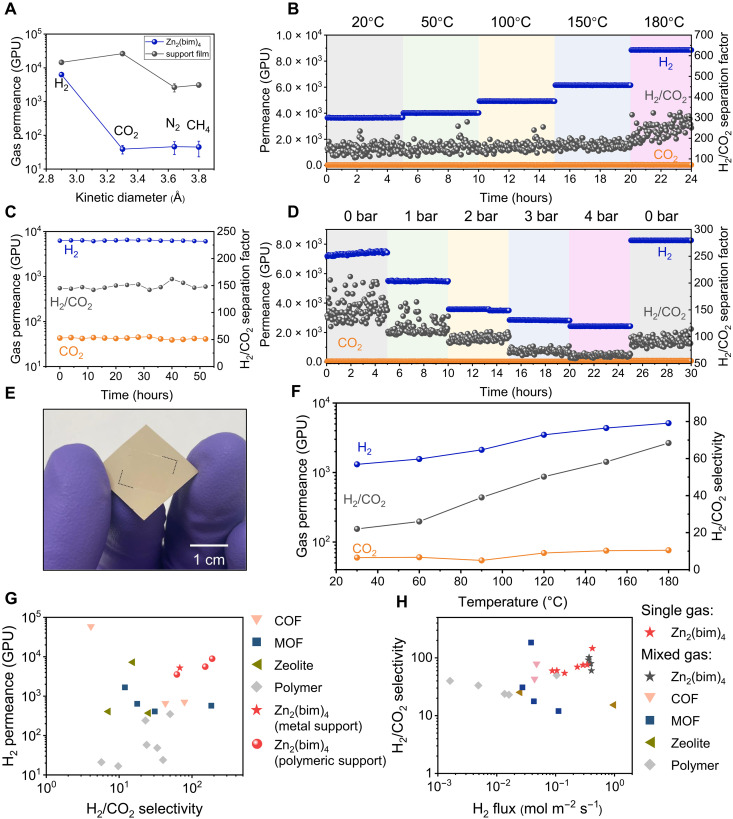
High-performance H_2_/CO_2_ separation from 2D Zn_2_(bim)_4_ membranes. (**A**) H_2_, CO_2_, N_2_, and CH_4_ permeances of the support film and the supported Zn_2_(bim)_4_ membrane (∆*P* of 1 bar). The error bar corresponds to the SD based on measurements from three separate membranes. (**B**) H_2_/CO_2_ separation performance with the temperature increasing from 20° to 180°C. (**C**) Zn_2_(bim)_4_ membrane separation performance for an equimolar H_2_/CO_2_ mixed feed (∆*P* of 1 bar). (**D**) H_2_/CO_2_ separation performance with ∆*P* increasing from 1 to 4 bar and then back to 1 bar. (**E**) Photograph of the Zn_2_(bim)_4_ membrane prepared on the polymeric PBI support. (**F**) The corresponding H_2_/CO_2_ separation performance as a function of temperature. Comparison of the H_2_/CO_2_ separation performance of Zn_2_(bim)_4_ membranes with the state-of-the-art membrane: (**G**) the H_2_ permeance and (**H**) the total H_2_ flux (reported under pressurized conditions, tables S3 and S4).

Single-gas permeation through 2D Zn_2_(bim)_4_ film yielded distinct transport rates for H_2_ and CO_2_ molecules with an average H_2_/CO_2_ ideal selectivity of 160 at 25°C under pressurized condition (∆P of 1 bar, Fig. 4A). The corresponding H_2_ permeance was large (6290 GPU), thanks to a selective layer that is only 4 nm thick. An equimolar H_2_/CO_2_ feed under pressurized conditions (∆P of 1 bar) was studied to understand transport in a mixed gas environment. A high separation performance was also obtained in the mixed feed with a H_2_ permeance of 5890 GPU and a H_2_/CO_2_ separation factor (SF) of 152 at 20°C (Fig. 4, B and C). When the temperature was increased to 180°C, H_2_ permeance increased to 8900 GPU, and H_2_/CO_2_ SF increased to 189 (Fig. 4B). Hydrothermal stability is a critical concern for practical H_2_ separation and purification. To evaluate this, we tested the membrane under humid feed conditions. As shown in fig. S26, the H_2_/CO_2_ SF decreased from 86 to 35 but was restored under dry conditions. This behavior is possibly due to interactions between the binding sites in the MOF and water molecules, where the adsorbed water facilitates CO_2_ transport, leading to a decrease in the H_2_/CO_2_ SF.

To understand the effect of defects on membrane performance, a Zn_2_(bim)_4_ membrane yielding an SF of 102 at a ∆P of 1 bar was further pressurized to a ∆P of 4 bar (Fig. 4D). A high SF of 62 was also obtained at a ∆P of 4 bar indicating a minor loss from of pinhole defects in the membrane. Moreover, when ∆P was reduced to 1 bar again, the SF increased to 81, indicating that pressurization did not damage the membrane. In comparison, state-of-the-art lamellar and polycrystalline MOF membranes are typically reported with a ∆P of 0. Often, performance is severely degraded at even a slight pressurization (∆P of 1 bar), indicating the presence of defects in these membranes. The robustness of the 2D Zn_2_(bim)_4_ film is attributed to continuous intergrown 2D film where grain-boundary defects are the only possible defective pathway.

The above membranes were prepared on the macroporous metal mesh. Zn_2_(bim)_4_ membranes could also be prepared on porous polymeric support, e.g., porous polybenzimidazole (PBI) hosting a pore size of 20 nm (*74*) (Fig. 4E). Membrane made in this way yielded an attractive H_2_ permeance of more than 5000 GPU and a selectivity of 65 at 180°C (Fig. 4F and fig. S27).

Thanks to the high performance of 2D Zn_2_(bim)_4_ membrane, the performance achieved here supersedes the performance of the state-of-the-art membranes for H_2_/CO_2_ separation, including those from the diverse material class of MOFs, COFs, polymers, and zeolites under pressurized feed condition critical for practical applications (Fig. 4, G and H, and tables S3 and S4). It is noteworthy that, compared to other MOF membranes with ultrahigh selectivity (>1000) tested under zero trans-membrane pressure (*36*, *65*), our work demonstrates enhanced performance over state-of-the-art membranes, particularly under pressurized conditions. Overall, the 2D Zn_2_(bim)_4_ film displayed strong separation performance for H_2_/CO_2_, delivering results that exceed those of current state-of-the-art membranes. In addition, pressurization did not damage the membrane, indicating the durability of the 2D Zn_2_(bim)_4_ film, further underscoring its potential for practical application.

## DISCUSSION

A first example of the 2D Zn_2_(bim)_4_ film with an infinite aspect ratio is demonstrated. Zn_2_(bim)_4_ is ideal for H_2_-selective transport controlled by the 4-MR aperture. The method developed in this study allowed the formation of a highly uniform, oriented, and crystalline film. As a result, extremely attractive H_2_/CO_2_ separation performance could be obtained under pressurized conditions. The advantage of this work is further highlighted by the fact that films were synthesized using extremely simple and user-friendly crystallization conditions (room temperature synthesis within minutes), improving environmental sustainability (use of water as solvent). The high performance of the film is attributed to the in-plane intergrowth, which eliminates pinhole defects. These findings will inspire the development of other promising MOF frameworks in the 2D film morphology for critical separation application.

## MATERIALS AND METHODS

### Chemicals

Zn(NO_3_)_2_·6H_2_O was purchased from Sigma-Aldrich. In addition, benzimidazole (bim) was obtained from Sigma-Aldrich. HCl (32 wt %) was purchased from Reactolab S.A. Poly(1-trimethylsilyl-1-propyne) (PTMSP) was obtained from ABCR. Poly(methyl methacrylate) (PMMA) was purchased from MicroChem. FeCl_3_ (97%) and Na_2_S_2_O_8_ were bought from Sigma-Aldrich. Cu foil (50 mm, 99.9%) was purchased from Strem. Toluene (AR) and methanol (AR) were obtained from Thermo Fisher Scientific. All the chemicals were used without further purifications. Si/SiO_2_ wafers were purchased from University Wafer. HOPG (ZYB quality, GRBS/2.0 × 10.0 × 10.0, mosaic spread: 0.8° to 1.2°) was purchased from ScanSens. The PBI-AM Fumion powder was purchased from Fumatech. Stainless-steel mesh (pore size 20 μm, part number #325X2300TL0014) was purchased from TWP Inc.

### Synthesis of the Zn_2_(bim)_4_ film for SEM and AFM

In a petri dish containing 18 ml of Zn_2_(NO_3_)_2_ aqueous solution at room temperature, various substrates—HOPG, CVD graphene on Cu foil, and graphene/Si/SiO_2_—were partially immersed. Subsequently, 2 ml of an aqueous solution of bim was added. After a predetermined duration, the substrates were carefully removed to stop the reaction. This procedure allowed us to distinguish the edge between the bare substrate and the Zn_2_(bim)_4_ film, facilitating the observation of thickness and contrast using SEM and AFM. Further details on how the concentration of the precursor solution and the reaction time influence the outcomes are discussed in note S1.

### Sample preparation of the Zn_2_(bim)_4_ film on graphene for TEM and SAED

First, we prepared the graphene sample lying on the TEM grid as reported before. Briefly, paraffin was melted and spin coated on top of the Cu-supported graphene. Then, the Cu-foil was etched using 10 wt % Na_2_S_2_O_8_ solution to obtain a paraffin-reinforced graphene. Then, it was washed with water three times and subsequently transferred to the TEM grid. The transferred film was annealed at 45°C for 2 days to strengthen the adhesion of graphene to the TEM grids. Last, the paraffin layer was removed by immersing the grid for two hours in three consecutive heptane baths and dried in the fume hood overnight.

Subsequently, the graphene on TEM grid was floated on the Zn(NO_3_)_2_ aqueous solution (18 ml, 6.94 mM) in a petri dish, followed by the addition of a bim aqueous solution (2 ml, 0.06 M) for 10 min. After that, the grid was removed to stop the reaction.

### Sample preparation of the Zn_2_(bim)_4_ film on graphene for GIXRD

First, we prepared graphene on a Si/SiO_2_ wafer as the substrate using PMMA for the graphene transfer. The PMMA solution was spin coated onto CVD-Graphene at 2000 rpm for 2 min. After drying overnight, the copper was etched away using a 10 wt % Na_2_S_2_O_8_ solution. The sample was then washed with water three times and subsequently transferred to the Si/SiO_2_ wafer. The transferred film was dried overnight in the fume hood and then baked in an oven at 150°C for 10 min to enhance attachment to the Si/SiO_2_ wafer. Subsequently, the PMMA layer was removed using fresh acetone, with three washing cycles of 1 hour each. Following this, the substrates were immersed in the precursor solution (6 mM Zn^2+^, 6.25 mM bim^−^) for 30 min. Any residual liquid on the surface was then gently wiped off from the edges using tissues.

### Synthesis of the Zn_2_(bim)_4_ membrane for gas separation

Initially, single-layer graphene was synthesized using a low-pressure CVD method with methane on copper foil, as reported in the literature (*75*). The process began with annealing the copper foil at 1077°C in a H_2_/Ar atmosphere for 60 min. This was followed by introducing a flow of CO_2_ (100 ml min^−1^) and H_2_ (8 ml min^−1^), each for 30 min, to cleanse the copper of any contaminants. Last, the single-layer graphene was grown on the prepared copper surface by using a mixture of CH_4_ (24 ml min^−1^) and H_2_ (8 ml min^−1^) for 30 min at a pressure of 460 mtorr.

After synthesizing the single-layer graphene, we used an O_2_ plasma treatment (MTI Plasma Cleaner, EQ-PCE-3, 13.56 MHz, 17 W) to create nanopores. This step was crucial for the application of the Zn_2_(bim)_4_ film as the selective layer in the membrane. We began by replacing the plasma chamber atmosphere with an O_2_ flow, achieving a pressure of approximately 50 mtorr. Then, a 6-s plasma etch was performed to transform the single-layer graphene into NG. Subsequently, we spin coated a PTMSP solution (1.25 wt %, in toluene) onto the NG. This was done at two speeds: first at 1000 rpm for 30 s, and then at 2000 rpm for another 30 s. The coated sample was left to dry overnight in a clean hood. To remove the graphene on the back side, we floated the PTMSP-coated graphene/Cu sample on a Na_2_S_2_O_8_ solution (10 wt %, aqueous) and then in deionized (DI) water, each for 5 min. We used lens tissue to clean the noncoated side until no black contaminants were visible on the tissue.

For etching and cleaning the Cu foil, a combination of FeCl_3_ (0.5 M aqueous solution), HCl (0.5 M aqueous solution), and DI water was used. The graphene/PTMSP film was then transferred onto the surface of a Zn(NO_3_)_2_ aqueous solution (18 ml, 6.94 mM) in a petri dish, followed by the addition of a bim aqueous solution (2 ml, 0.06 M). After 10 min of growth, the resulting Zn_2_(bim)_4_/graphene/PTMSP film was transferred onto a W support for gas permeation testing. The protocols for MOF samples on polymeric supports are similar, with 3 wt % PTMSP solution being used for the coating layer and porous PBI support as the substrate (for details, see note S5).

### Characterizations and measurements

SEM measurements were performed on a Teneo SEM instrument operating at 1 kV and working distances of 5 to 8 mm. The powder x-ray diffraction data were collected at a Bruker D8 Discover diffractometer with a Lynxeye XE detector, operated at 40 kV and 400 mA under Cu Kα radiation (λ = 1.5406 Å) at ambient temperature and pressure. Bright-field TEM images and SAED images were obtained with a FEI Tecnai G2 Spirit Twin microscope operated at 120 kV.

AFM and KPFM measurements were recorded on a Bruker MultiMode 8 AFM instrument. For KPFM measurements, the SCM-PIT-V2 probe was used and calibrated with a standard sample of HOPG. XPS data were obtained on an Axis Supra instrument (Kratos Analytical) using the monochromated K x-ray line of an aluminum anode. Synchrotron GIXRD data were obtained at beamline BM01, Swiss-Norwegian beamline (SNBL), at the European Synchrotron Radiation Facility (ESRF) at wavelengths of 1.04157 Å (*76*), and data analysis was with the tools developed at the beamline as discussed in the previous work (*69*) (for details, see note S4).

We tested the gas separation performance of the metal-mesh supported membranes using a custom-built permeation setup. During the tests, we maintained the pressure on the feed side between 1 and 5 bar, while the permeate side was kept at 1 bar. All measurements were conducted after reaching a steady state, with argon used as the sweep gas. The membranes were carefully sealed using a stainless-steel gasket to ensure accurate results. For analyzing the composition of the permeate, we used an online Hiden Analytical HPR-20 mass spectrometer.

The permeances *J_i_* of gas *i* was calculated as follows

$$J_{i}={Χ}_{i}/\left(A\times ∆P_{i}\right)$$ (1)

In this formula, ${Χ}_{i}$ represents the molar flow rate of component *i* across membrane area *A*, and $∆P_{i}$ denotes the transmembrane pressure difference for component *i*. The selectivity α*_ij_*, between two gases *i* and *j* (where *i* is the faster permeating gas), is calculated using the following equation

$$\alpha_{\mathrm{ij}}=J_{i}/J_{j}$$ (2)

## Supplementary Materials

### This PDF file includes:

Supplementary Notes S1 to S5 Figs. S1 to S27 Tables S1 to S5 References
