# Supplementary material for: High-performance H2/CO2 separation from 4-nm-thick oriented Zn2(benzimidazole)4 films
Source: Sci Adv. 2024 Dec 13;10(50):eads6315. doi: 10.1126/sciadv.ads6315 (PMC11641003; doi:10.1126/sciadv.ads6315)
Supplement: Supplementary file 1 — Supplementary Notes S1 to S5 Figs. S1 to S27 Tables S1 to S5 References [file sciadv.ads6315_sm.pdf]

Science Advances
AAAS

Supplementary Materials for

High-performance H_2/CO_2 separation from 4-nm-thick oriented Zn_2(benzimidazole)$_4$ film

Shuqing Song *et al.*

Corresponding author: Kumar Varoon Agrawal, kumar.agrawal@epfl.ch

Sci. Adv. **10**, eads6315 (2024)
DOI: 10.1126/sciadv.ads6315

This PDF file includes:

Supplementary Notes S1 to S5
Figs. S1 to S27
Tables S1 to S5
References

Supplementary Notes:

Note S1. Effect of the concentration and ratio of Zn^{2+} and bim^- for the $Zn_2(bim)_4$ film growth on graphene.

To optimize the precursor solution and metal/linker ratio for $Zn_2(bim)_4$ film growth, we experimented with various precursor concentrations on Cu-supported graphene samples (as shown in Fig. S1). Initially, we fixed the growth time at 10 minutes and varied the precursor solution to observe changes in film growth. Starting with the most dilute precursor solution (Zn^{2+}: 1.6 mM, bim^-: 1.5 mM, Zn/bim ratio of 1.04), we observed no film growth on the graphene surface (Fig. S1A). When we doubled the concentration of the precursor solution (Zn^{2+}: 3.1 mM, bim^-: 3.0 mM), the film began to grow, but discontinuously (Fig. S1B). Increasing the precursor solution concentration further led to continuous film growth (Figs. S1E-I), with Zn^{2+} concentration ranging from 4.7 mM to 9.4 mM, bim^- concentration from 4.5 mM to 9.0 mM, and Zn/bim ratios from 0.5 to 2.1. Altering the metal/linker ratio (Zn^{2+} concentration from 3.1 mM to 12.5 mM, bim^- concentration from 12.0 mM to 3.0 mM, Zn^{2+}/bim^- ratio from 0.25 to 4) resulted in the films growing discontinuously again (Figs. 1C-D). Thus, we determined that the optimal metal/linker ratio for film growth is 1.04. Upon further increasing the precursor concentration while maintaining a fixed metal/linker ratio of 1.04 (Zn^{2+} from 12.5 mM to 18.8 mM, $bim^-$from 12.0 mM to 18.0 mM), we noted the aggregation of powder on the film (Figs. S1J-K).

Note S2. Indexation of electron diffraction data from $Zn_2(bim)_4$ and Graphene patterns.

The electron diffraction data of $Zn_2(bim)_4$/Graphene film were indexed based on the symmetry and calculation of each diffraction dot, and compared with the theoretical data. The *d*-spacing and angle between each lattice plane could be calculated by the following equations:

Graphene is a two-dimensional material with a hexagonal symmetry, and the *d*-spacing can be calculated by:

$$\frac{1}{d^2} = \frac{3}{2}\left(\frac{h^2 - hk + k^2}{a^2}\right)$$

Meanwhile, the theoretical angles between lattice planes of graphene were calculated by:

$$\cos\Phi = \frac{h_1h_2 + k_1k_2 - \frac{1}{2}(h_1k_2 + h_2k_1)}{\sqrt{(h_1^2 + k_1^2 + h_1k_1)(h_2^2 + k_2^2 + h_2k_2)}}$$

For $Zn_2(bim)_4$, which is a monoclinic system,

$$\frac{1}{d^2} = \frac{1}{\sin^2\beta}\left(\frac{h^2}{a^2} + \frac{k^2\sin^2\beta}{b^2} + \frac{l^2}{c^2} - \frac{2hl\cos\beta}{ac}\right)$$

And the theoretical angles between lattice planes of $Zn_2(bim)_4$ were calculated by:

$$\cos\Phi = \frac{h_1h_2 + k_1k_2 + l_1l_2}{\sqrt{(h_1^2 + k_1^2 + l_1^2)(h_2^2 + k_2^2 + l_2^2)}}$$

To determine the zone axis in the diffraction pattern, it was aligned perpendicular to all the lattice planes, which was calculated by:

$$h_{ZA} \times h_1 + k_{ZA} \times k_1 + l_{ZA} \times l_1 = 0$$

where h_{ZA}, k_{ZA} and l_{ZA} indicate the lattice plane parameters of the zone axis and the h_1, k_1 and l_1 represent those of diffraction patterns.

For the XRD data, the equations for d-spacing and theoretical angles between lattice planes of the $Zn_2(bim)_4$ were the same.

Note S3. Calculation of lattice mismatch between $Zn_2(bim)_4$ and graphene.

The lattice mismatch is calculated by the following equation,

$$\text{Lattice mismatch} = \frac{d_{Zn_2(bim)_4} - d_{graphene}}{\mathrm{d}_{graphene}} \times 100\%$$

Our analysis, based on electron diffraction and GIXRD data, initially focuses on the lattice parameters of $Zn_2(bim)_4$ when applied to graphene, as well as the orientation relationships between the lattices of $Zn_2(bim)_4$ and graphene. We observed that the lattice parameters of $Zn_2(bim)_4$ are notably larger than that of graphene. Consequently, in our calculations, we employ a graphene superlattice (supercell) for comparison (Fig. S9). This approach allows for more accurate analysis by accommodating the differences in lattice size between $Zn_2(bim)_4$ and graphene.

Note S4. GIXRD measurement and analyses of $Zn_2(bim)_4$/graphene films.

For the GIXRD measurements, we synthesized $Zn_2(bim)_4$ films on graphene, which had been transferred onto SiO_2/Si wafers (Fig. S4). The MOF film synthesis was carried out under consistent condition: 6 mM Zn^{2+}, 6.25 mM bim^-. To create a film thick enough for GIWAXS testing, we performed 30 minutes on the graphene. This process resulted in a $Zn_2(bim)_4$ film approximately ~12 nm thick (Fig. S10). Data collection was conducted at BM01 Swiss-Norwegian Beamline (SNBL) at the European Synchrotron Radiation Facility (ESRF), using a PILATUS2M multipurpose detector. The GIWAXS employed a wavelength of 1.04157 Å. During testing, the X-ray beam, measuring 200 μm by 80 μm, was horizontally aligned with the samples.

(a) X-ray beam calibration

Prior to conducting the measurements, a standard LaB6 sample was utilized for X-ray beam calibration. Subsequently, each sample was positioned horizontally. We then aligned each sample to ensure that the X-ray was partially obstructed by the sample. This alignment was achieved through a combined use of ω-scan and z-scan.

(b) Optimization of measurement geometry

Prior to gathering diffraction data, we initially conducted a precise ω-scan. This allowed us to collect a series of diffraction data sets. After each set, we evaluated the diffraction quality to determine the optimal measurement geometry for subsequent data collection.

For aligning the measurement geometry of $Zn_2(bim)_4$/Graphene, we performed a sequence of ω-scans. Each scan had a step size of 0.02° and lasted 10 seconds, covering a range from -2° to 2°. This data was then analyzed to identify the most effective diffraction geometry. Through this analysis, an ω angle of 0.02° was determined to be the most suitable. Additionally, the setup maintained a distance of 300 mm between the sample and the detector.

(c) Collection of diffraction data

For the measurement of $Zn_2(bim)_4$/graphene, the diffraction data of $Zn_2(bim)_4$ was collected where grazing incidence was applied at 0.02°, and collecting time was 10 minutes, with the distance of 300 mm from the sample to the detector.

(d) Analyses of X-ray diffraction data

In the process of indexing diffraction data, we initially read the *q* value and the angle for each diffraction dot. This step yielded corresponding *d*-spacing values and angles. Next, we calculated the theoretical *d*-spacing values and angles for all lattice planes of the samples. The final step involved comparing these experimental results with the theoretical data. Through this comparison, we were able to confirm the

corresponding Miller indices. Consequently, this indexing allowed us to accurately determine the orientation of the sample.

Experimental q value was calculated by the following equation,

$$q = \frac{4\pi \sin\theta}{\lambda}$$

Experimental d spacing value was calculated by the following equation,

$$d = \frac{\lambda}{2\sin\theta} = \frac{2\pi}{q}$$

Theoretical d spacing value was calculated by the following equations. For $Zn_2(bim)_4$, which is a monoclinic system,

$$\frac{1}{d^2} = \frac{1}{\sin^2\beta}\left(\frac{h^2}{a^2} + \frac{k^2\sin^2\beta}{b^2} + \frac{l^2}{c^2} - \frac{2hl\cos\beta}{ac}\right)$$

Note S5: Protocols for polymeric support preparation.

The protocols for preparing the polymeric support are detailed in our previous work (*74*). 8 wt% polymer coating solution was prepared by stirring PBI-AM in NMP. The solution was used without any further treatment. A casting knife with a gap of approximately 250 μm was used to cast the solution onto the stainless-steel mesh. After casting, the mesh was placed in a water coagulation bath at 60°C and left overnight. The supports were then washed with deionized water and allowed to dry at room temperature. Finally, the PBI-AM coated mesh was heated at 330°C for 8 hours.

Supplementary Figures:

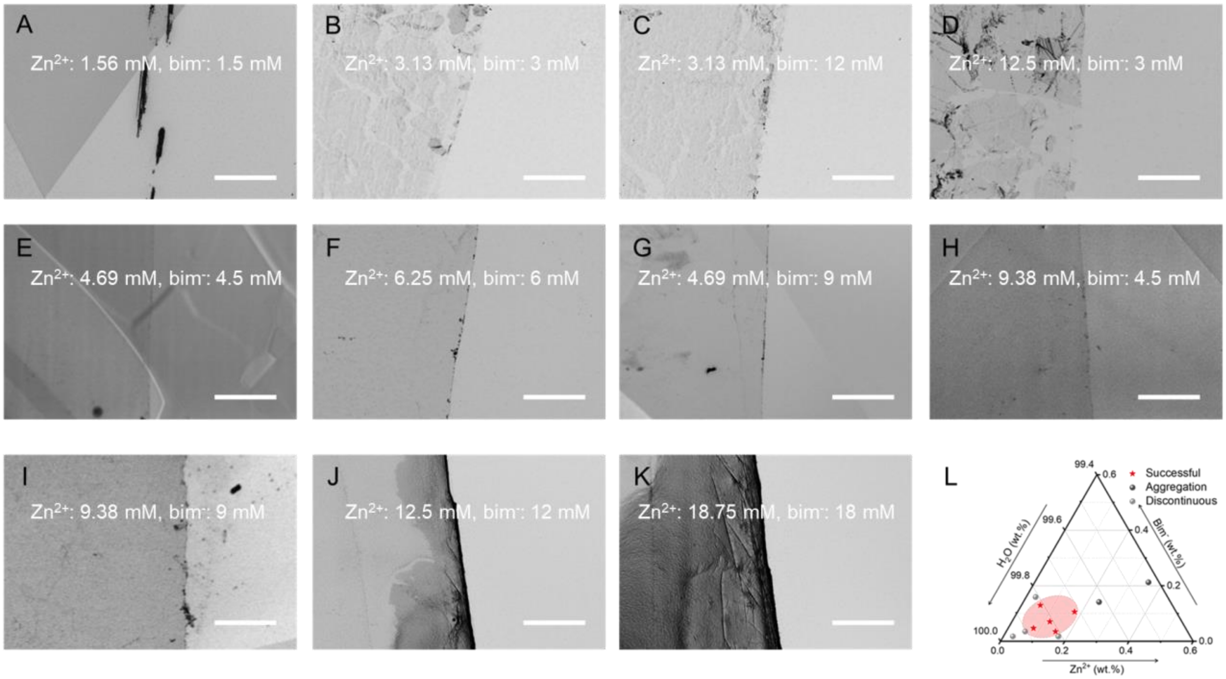

Fig. S1. SEM images of the $Zn_2(bim)_4$ film on CVD synthesized single-layer graphene on Cu foil under different precursor concentration with a fixed growth time of 10 minutes. (scale bar = 5 μm) (A) Zn^{2+}: 1.6 mM and bim^-:1.5 mM, (B) Zn^{2+}: 3.1 mM and bim^-:3 mM, (C) Zn^{2+}: 3.1 mM and bim^-:12.0 mM, (D) Zn^{2+}: 12.5 mM and bim^-:3.0 mM, (E) Zn^{2+}: 4.7 mM and bim^-:4.5 mM, (F) Zn^{2+}: 6.2 mM and bim^-:6 mM, (G) Zn^{2+}: 4.7 mM and bim^-:9.0 mM, (H) Zn^{2+}: 9.4 mM and bim^-:4.5 mM, (I) Zn^{2+}: 9.4 mM and bim^-:9.0 mM, (J) Zn^{2+}: 12.5 mM and bim^-:12.0 mM, (K) Zn^{2+}: 18.8 mM and bim^-:18.0 mM, and (L) the summary for the film forming condition of different precursor concentration. Grey spheres indicate the precursor concentration that can form discontinuous film, corresponding to panels (A-D), red stars with highlighted area show the concentration scale which can form continuous film, corresponding to panels (E-I), and the black spheres indicate the aggregation occurs under this concentration, corresponding to panels (J-K).

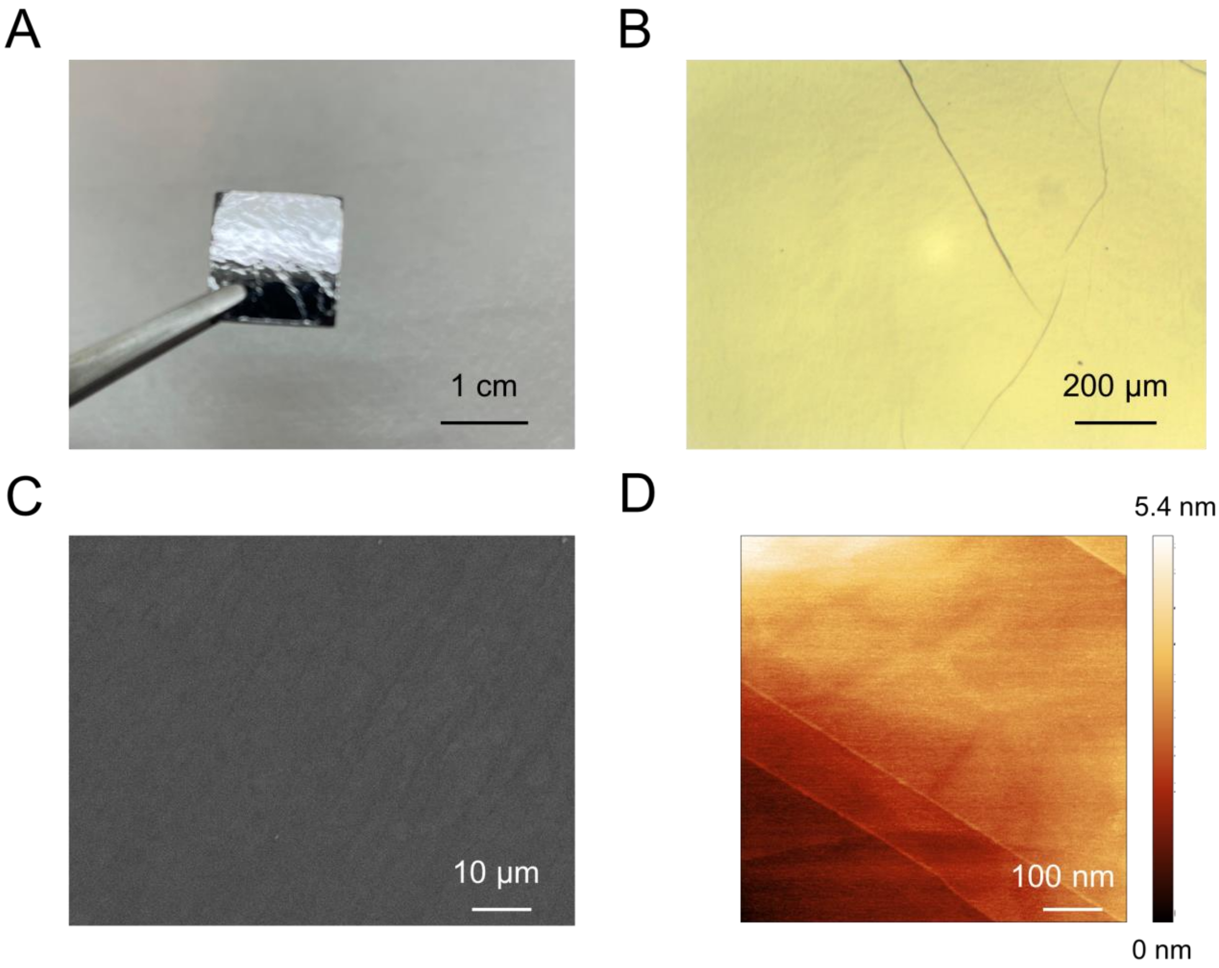

Fig. S2. Morphologies of the highly oriented pyrolytic graphite (HOPG). (A) Photograph, (B) optical microscopy image, (C) SEM, and (D) AFM images.

***Note:** Microscopy images of HOPG reveal a clear and smooth surface, making it an ideal substrate for the in-plane epitaxial growth of MOF films. It is important to note that the wrinkles observed on the surface are due to varying graphene layers, which form during the peeling process for the reuse of HOPG.

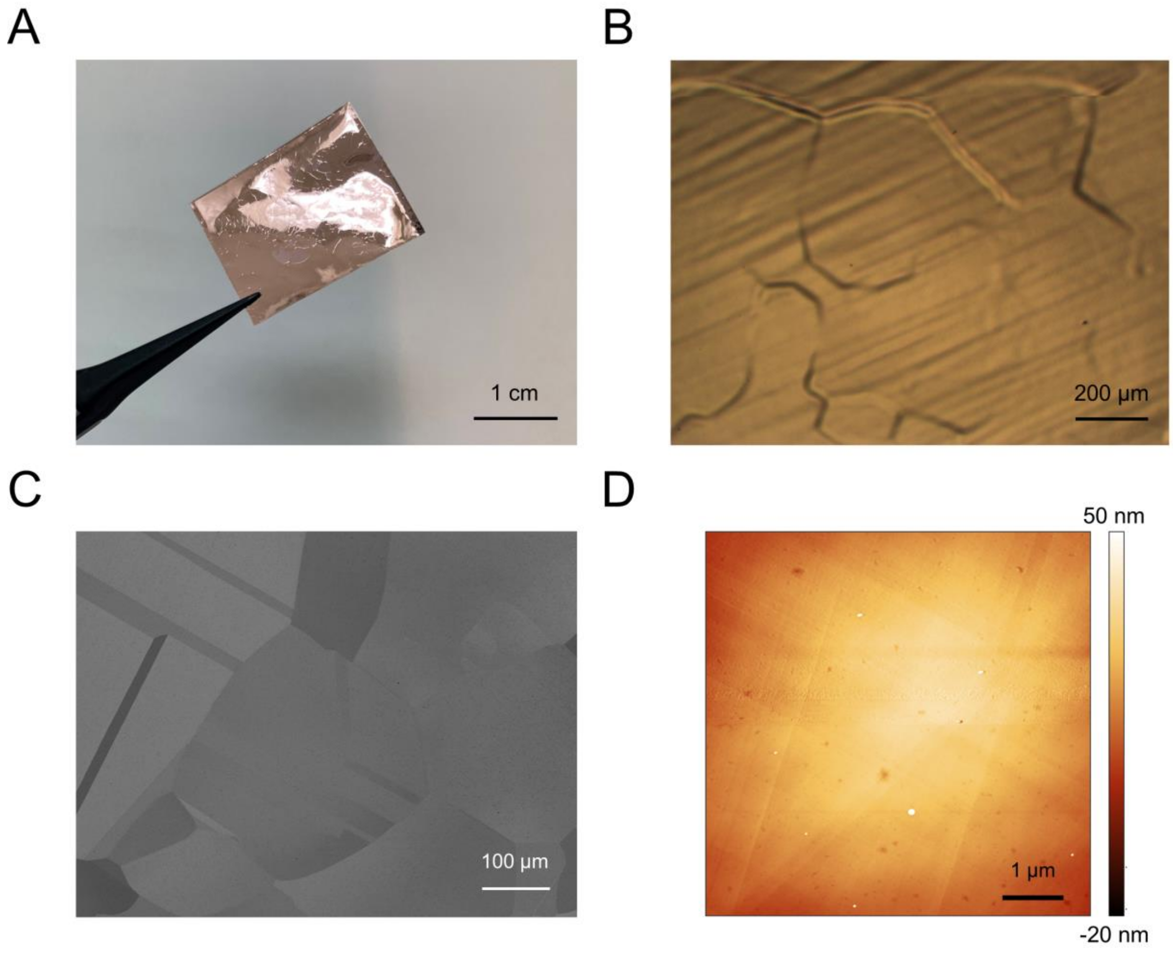

Fig. S3. Morphologies of the Chemical Vapor Deposition (CVD) synthesized single-layer graphene on Cu foil. (A) Photograph, (B) optical microscopy image, (C) SEM and (D) AFM images.

***Note:** In the CVD-synthesized graphene on Cu foil, the Cu grains are visible on the surface, with sizes ranging from 300 to 600 μm. Due to the lattice registry between the MOF and graphene (Fig. 2C), the large grain size and ultrasmooth surface of the substrate further facilitate the epitaxial growth of the top MOF layer.

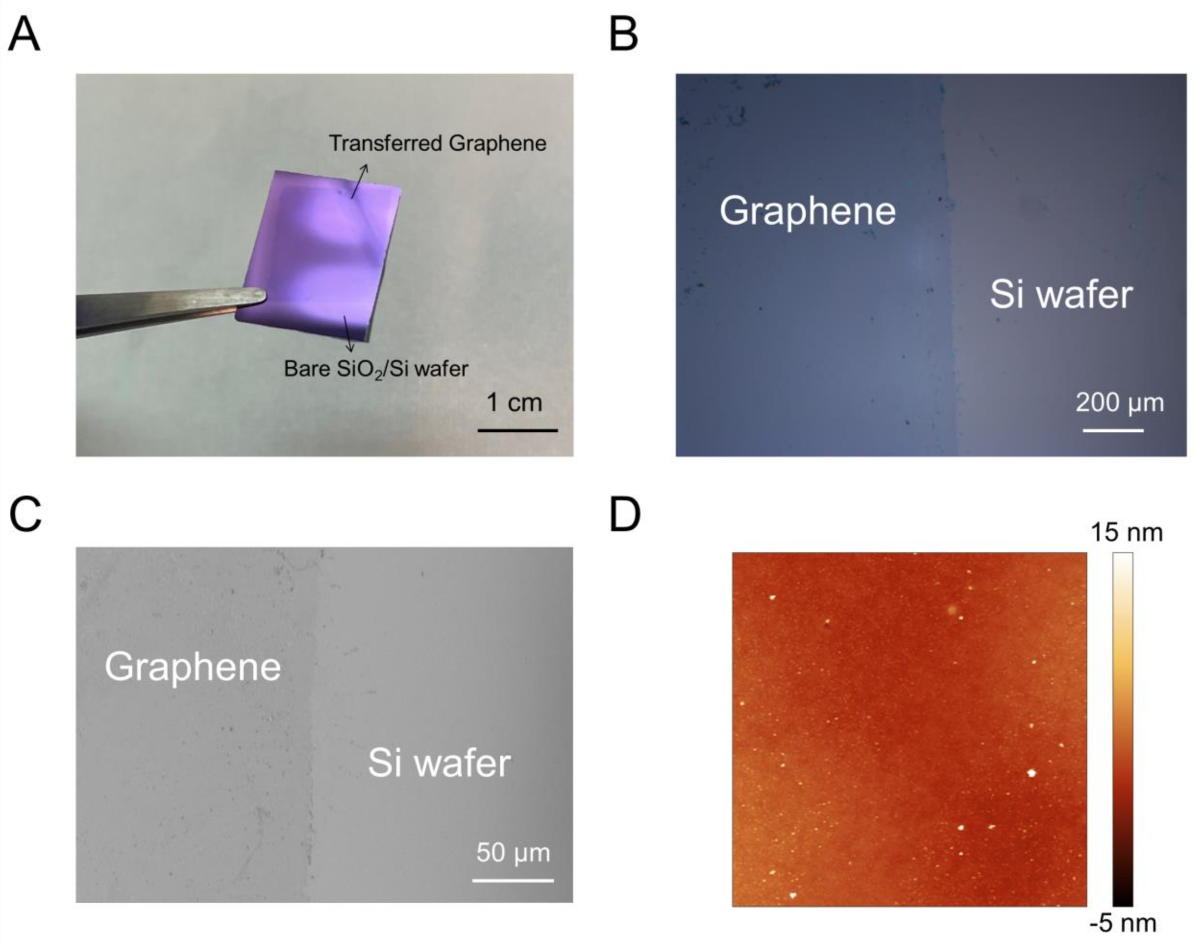

Fig. S4. (A) Photograph, (B) optical microscopy image, (C) SEM and (D) AFM images of the Si wafer-supported single-layer graphene.

***Note:** The interface between the graphene and the Si wafer is clearly visible in the images, emphasizing the quality of the transferred graphene layer. Following the PMMA removal process, the graphene maintains a mostly clean surface, with only minimal residue observed. This smooth, nearly contamination-free surface provides an excellent substrate for MOF growth and was used for the GIWAXS test.

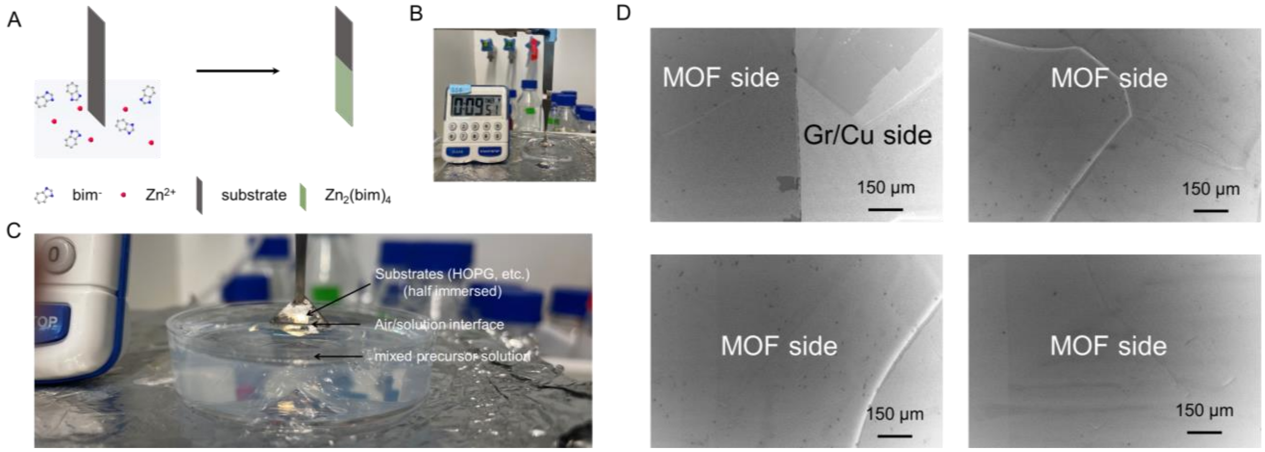

Fig. S5. The $Zn_2(bim)_4$ film synthesis process. (A) Schematic of the $Zn_2(bim)_4$ film growth on the substrates for SEM and AFM test. (B) The experimental set-up with a tweezer mounting the substrate half immersed into the precursor solution. (C) The phenomenon with a reaction time of 10 min. (D) SEM images of the $Zn_2(bim)_4$ film growing on Cu-supported graphene.

***Note:** The air/solution interface is visible, allowing for the observation of the film/substrate contrast. The bulk solution remains homogeneous and transparent with a slight milky appearance, indicating that nucleation was suppressed under ultra-dilute precursor conditions. Within this synthesis configuration, the film edge forms at the air/solution interface.

SEM images were acquired over a large sample area, with a lateral size of approximately 1.3 mm. A distinct interface between the MOF and bare graphene is visible in panel (D). We then imaged regions further along the side of the MOF film and captured three additional images. These images confirm a continuous film morphology, with no cracks or defects observed across this large area.

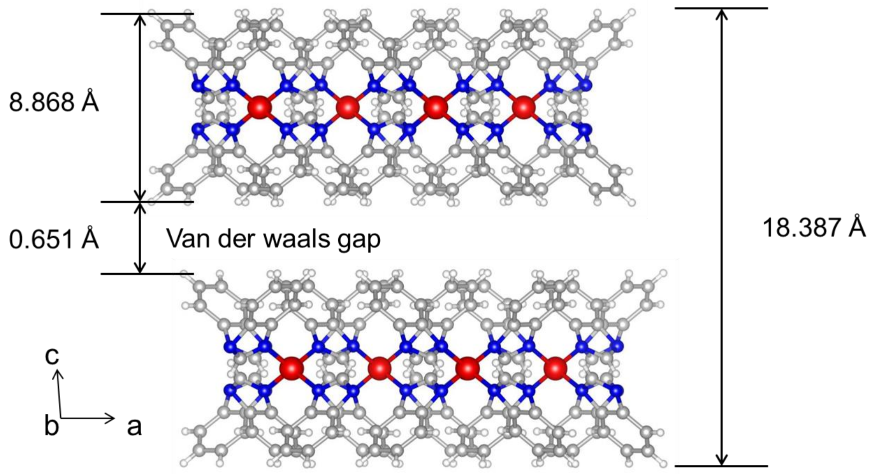

Fig. S6. The structure of $Zn_2(bim)_4$. Along *c* out-of-plane direction, each unit-cell contains 2 sublayers with a total thickness of 18.387 Å, including 1 van-der waals gap of 0.651 Å.

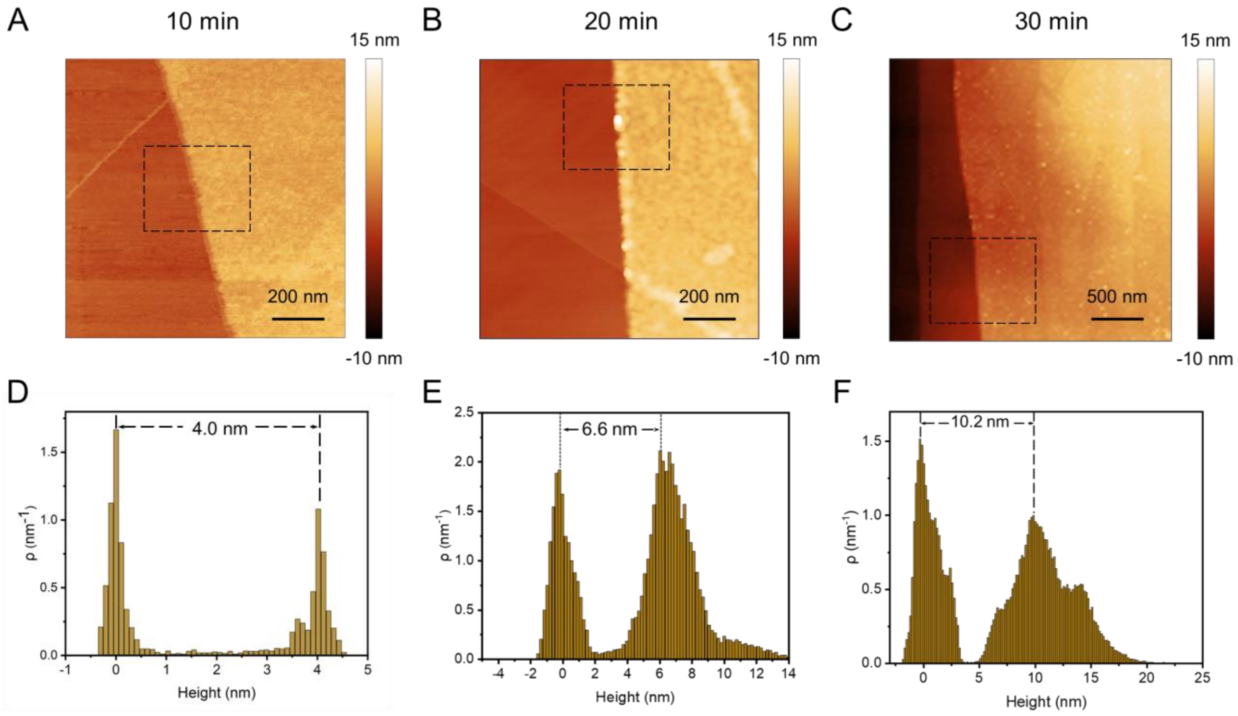

Fig. S7. AFM images and thicknesses of $Zn_2(bim)_4$ film on HOPG with different growth time. (A) 10 min, (B) 20 min, and (C) 30 min, and the corresponding height profile (D-F).

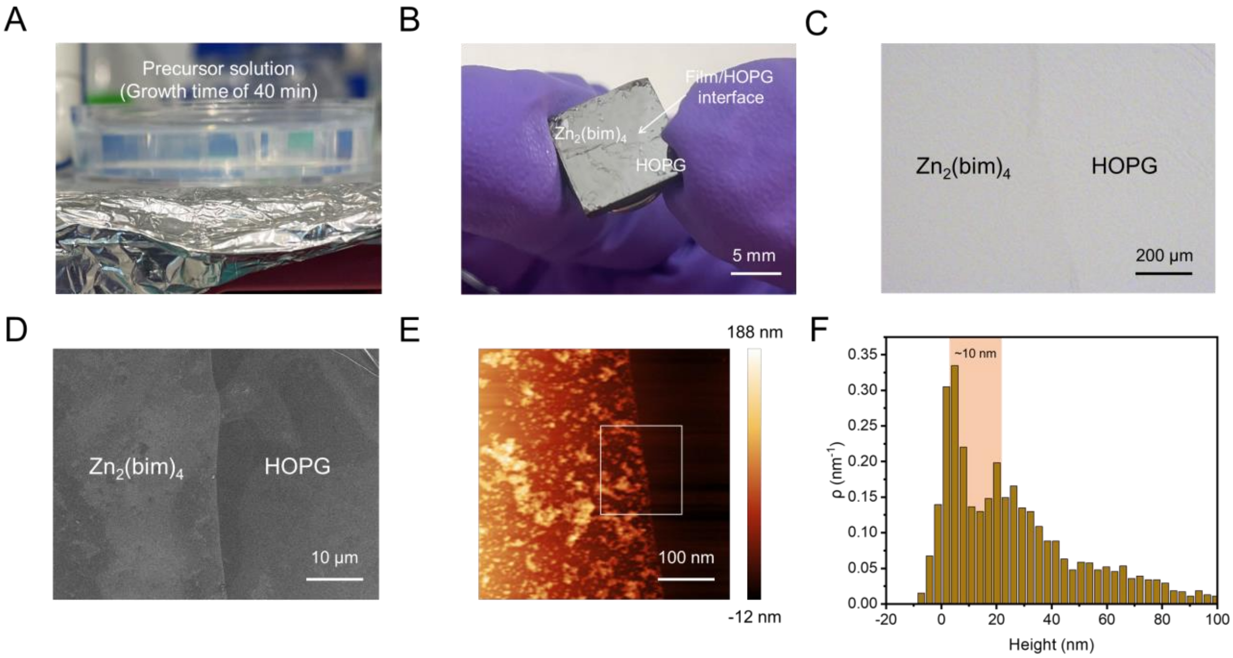

Fig. S8. The mixed precursor solution state and morphologies of the $Zn_2(bim)_4$ film on HOPG with a growth time of 40 minutes. (A) Photograph of the solution state in the bulk mixed precursor solution. (B) Photograph, (C) optical microscopy, (D) SEM and (E) AFM images of the resulting $Zn_2(bim)_4$ film on HOPG substrate. (F) The corresponding height histogram of the white circled area in (E).

***Note:** After a growth time of 40 minutes, the mixed precursor solution becomes noticeably powdery (Fig. S8A). This suggests notable nucleation within the solution, leading to powder easily adhering to the substrate surface. Consequently, the resulting MOF film is visible, with a distinct interface between the film and the HOPG substrate (Fig. S8B). However, analysis using optical microscopy (Fig. S8C), SEM (Fig. S8D), and AFM (Fig. S8E) reveals numerous powder particles on the $Zn_2(bim)_4$ film side. Notably, the film thickness does not show a notable increase (Fig. S8F).

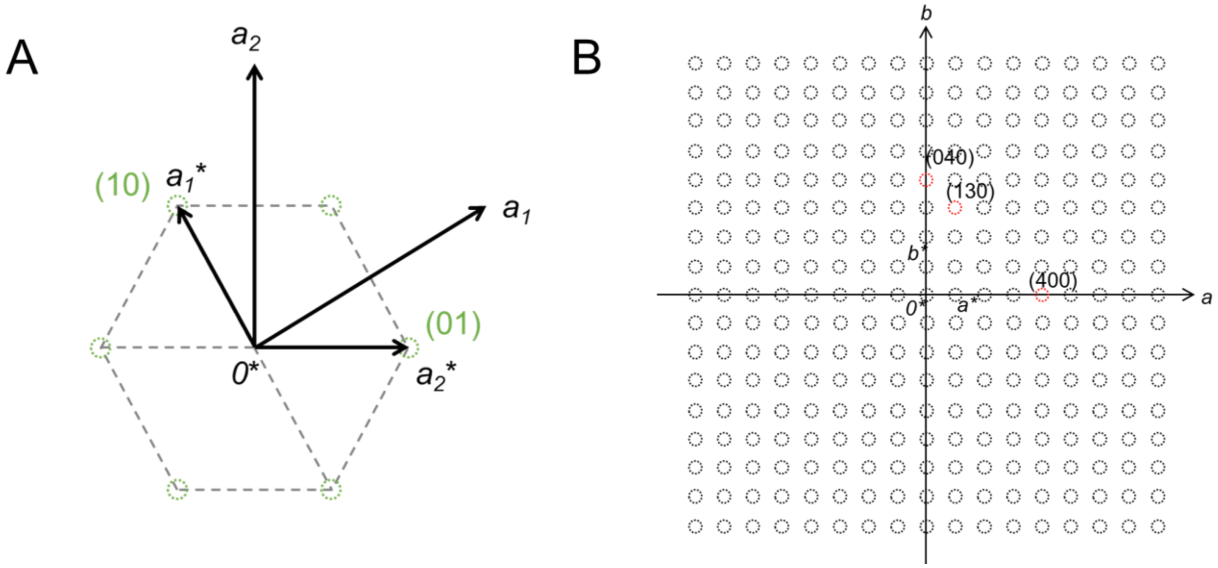

Fig. S9. Comparison of the superlattice of graphene and $Zn_2(bim)_4$. (A) illustration of reciprocal lattice of graphene, (B) illustration of reciprocal lattice of $Zn_2(bim)_4$, with zone axis of [002].

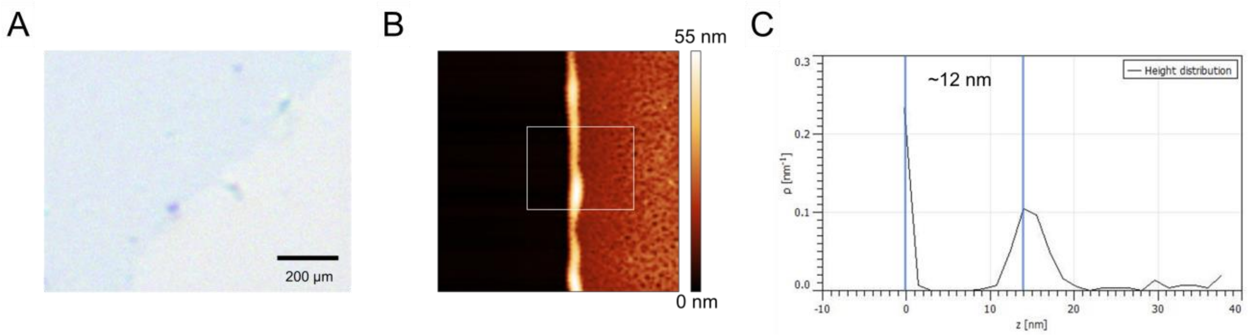

Fig. S10. $Zn_2(bim)_4$ on graphene/SiO_2/Si Sample for synchrotron GIWAXS test. (A) Optical microscopy image, (B) AFM image and (C) height profile of the sample.

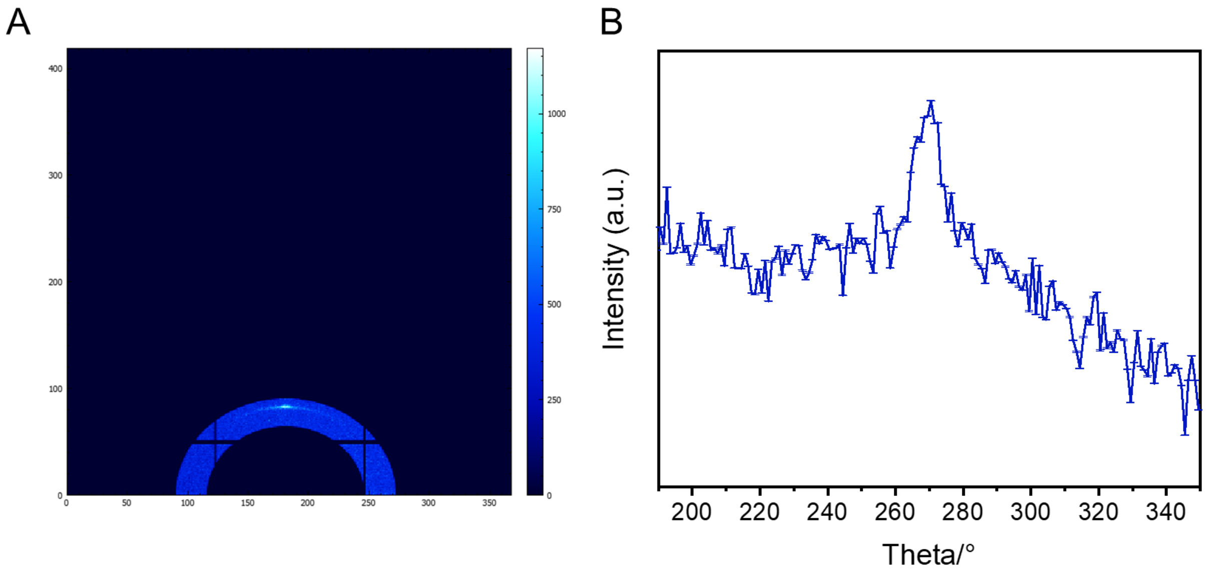

Fig. S11. (A) GIWAXS pattern of $Zn_2(bim)_4$ with integration azimuthally along the 002 peak. (B) Integrated azimuthal profile of the 002 peak.

***Note:** The preferential orientation can be quantified by integrating the GIWAXS pattern on the 2D detector across different regions. In this configuration, we focused on the intensity of the 002 peak. In panel b, the azimuthal angle χ at exactly 90° shows the strongest signal in the GIWAXS pattern, indicating a preferential orientation of the 002 planes in the out-of-plane direction.

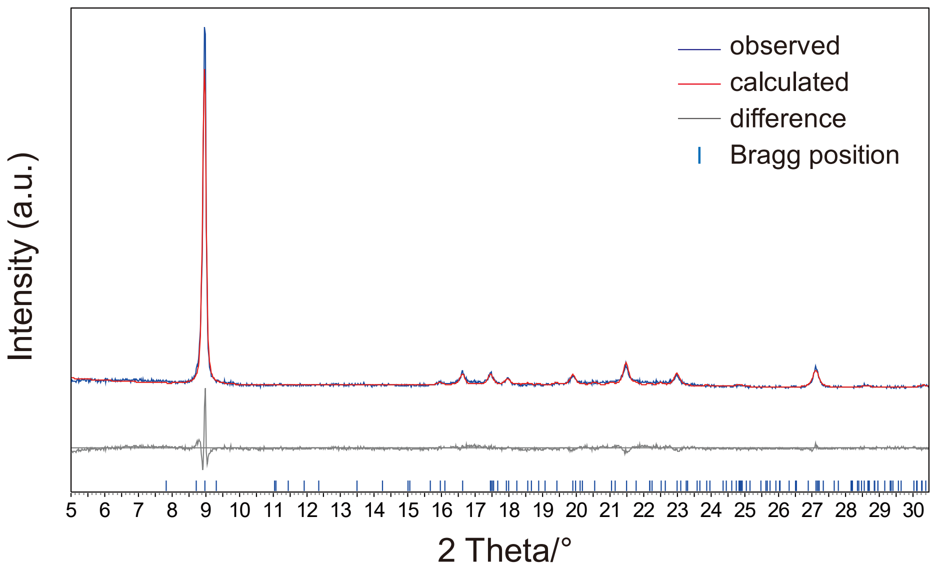

Fig. S12. Rietveld refinement of the patterns from the scrapped powder with the simulated $Zn_2(bim)_4$ pattern.

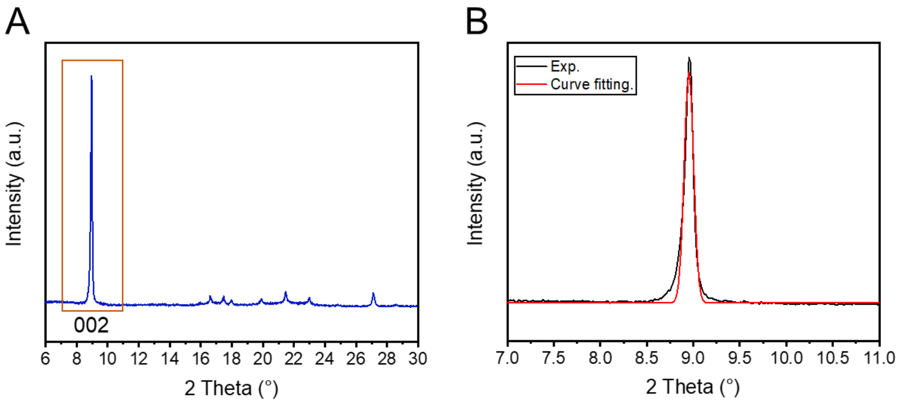

Model	Gaussian
Equation	y = y0 + A/(w*sqrt(pi/(4*ln(2)))) * exp(-4*ln(2)*(x-xc)^2/w^2)
Plot	Exp.
y0	2.87634 ± 0.04647
xc	8.9521 ± 4.3145E-4
A	16.29095 ± 0.1212
FHWM	0.11889 ± 0.00102
Reduced Chi-Sqr	2.93146
R2	0.9521
R2 after adjustment	0.952

Fig. S13. (A) High-resolution XRD data and (B) fitting curve from the peeled off film.

***Note**: We employed Scherrer analysis for the strongest 002 peak.

$$\tau = \frac{K\lambda}{\beta cos\theta}$$

The K factor, a constant dependent on the sample shape, is 0.94 in this case. θ represents the Bragg angle, β is the full width at half maximum (FWHM) of the peak (in radians), and λ is the X-ray wavelength (in nm). Based on these parameters, we calculated the equivalent crystallite size to be approximately 697 nm, consistent with the size of the 2D grains determined by electron diffraction. This suggests that the MOF film has a highly ordered structure.

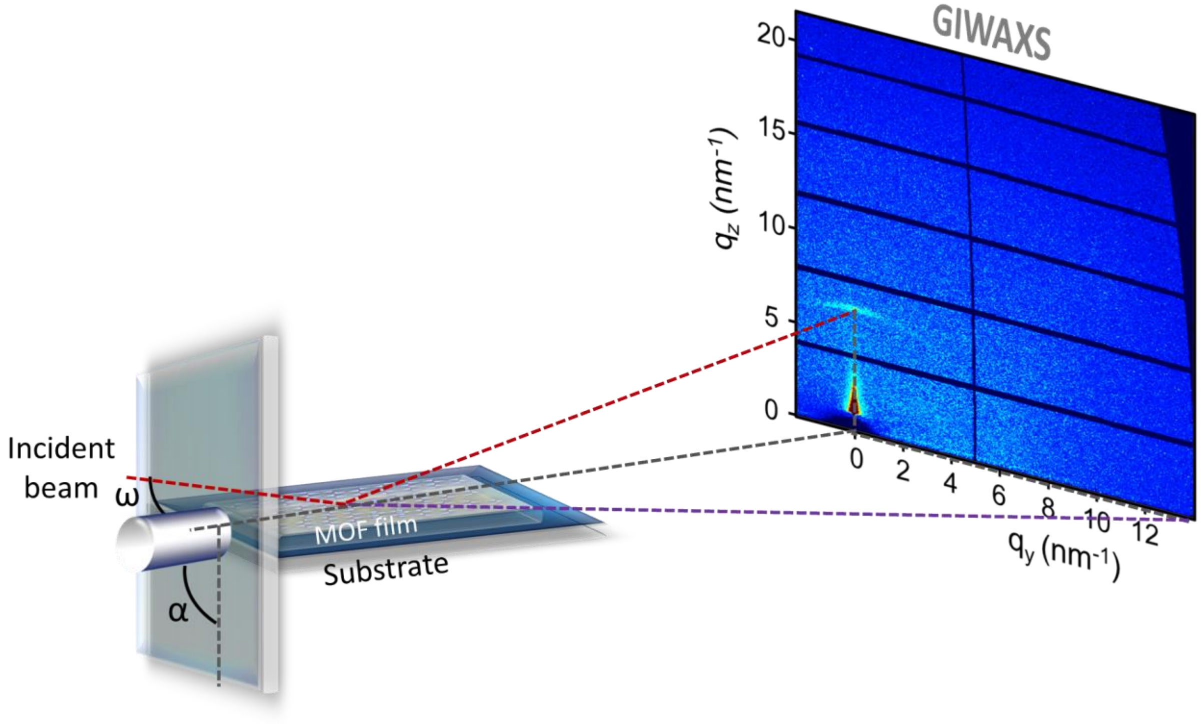

Fig. S14. Scattering geometry of GIWAXS. The incident beam (highlighted in dark red) is directed towards the sample at an incident angle denoted as α. This angle is adjusted by rotating the substrate to a certain degree, denoted as ω. In this configuration, the incidence angle (α) was determined by calculating the angle difference between the substrate surface and the beam (Δω).

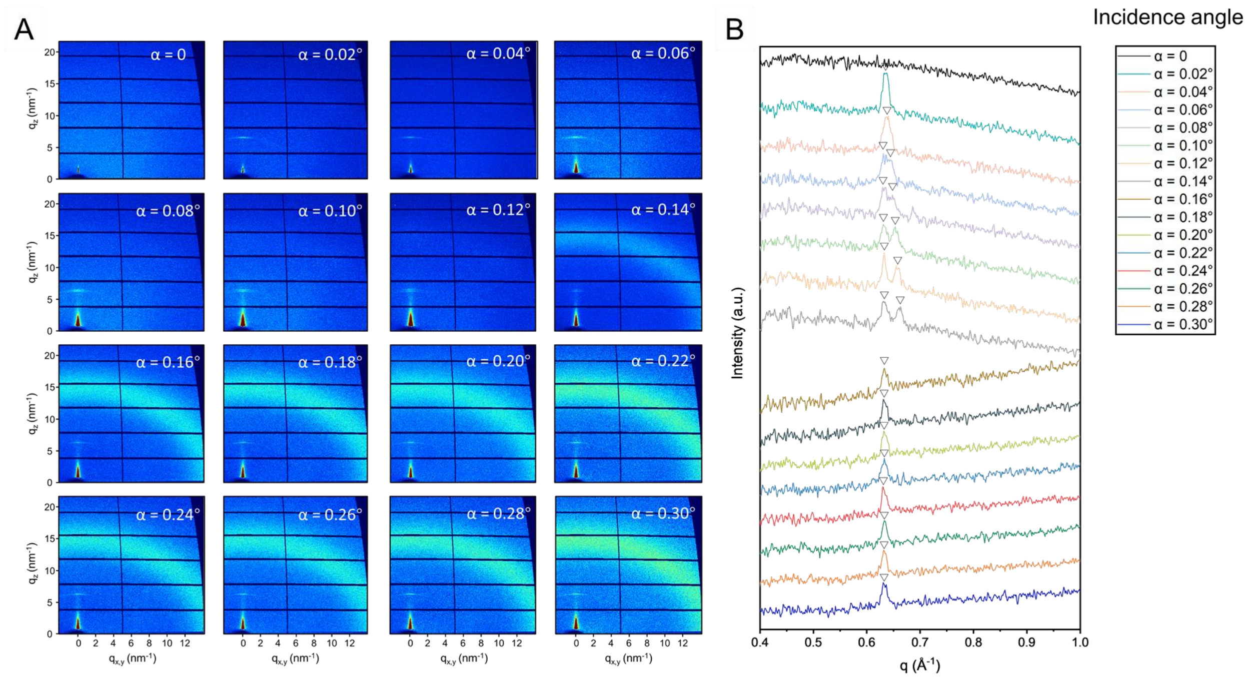

Fig. S15. (A) GIWAXS patterns of $Zn_2(bim)_4$ on graphene/SiO_2/Si wafer of incidence angles (α) from 0.02° to 0.3°. (B) The normalized intensity integrated from panel a, with the peak for 002 indicated by triangles.

***Note:** From the outset, we observe that at an incidence angle (α) ranging from 0.02 to 0.06°, the 002 peak manifests as a single entity. As the incidence angle extends beyond 0.08°, the peak begins to bifurcate into two distinct peaks. At an increased incidence angle of 0.14°, the discrepancy between the two 002 peaks becomes more pronounced. Beyond an incidence angle of 0.16°, the peaks once again coalesce into a single entity. Concurrently, as depicted in panel a, beginning at an incidence angle of 0.14°, the emergence of a robust, broad peak corresponding to amorphous SiO_2 is evident. This occurrence correlates with the deeper penetration of the beam as the incidence angle escalates.

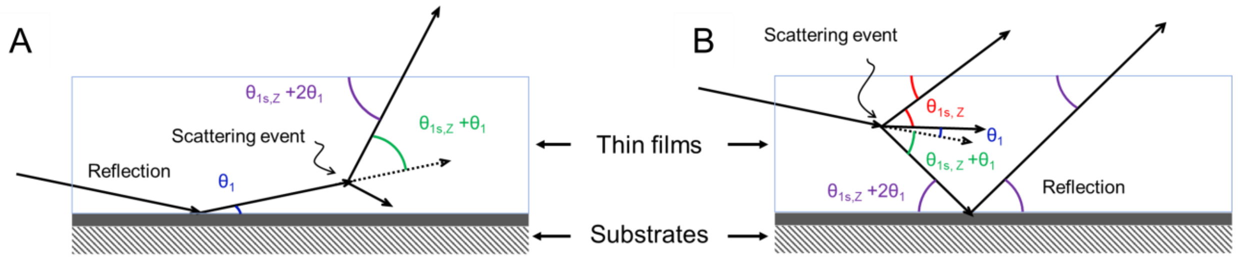

Fig. S16. Schematic of the double-peak effect, showing the ray tracing for the two equivalent geometric scenarios, (A) where the beam is first reflected and then scattered, and (B) when the direct beam is scattered and then reflected.

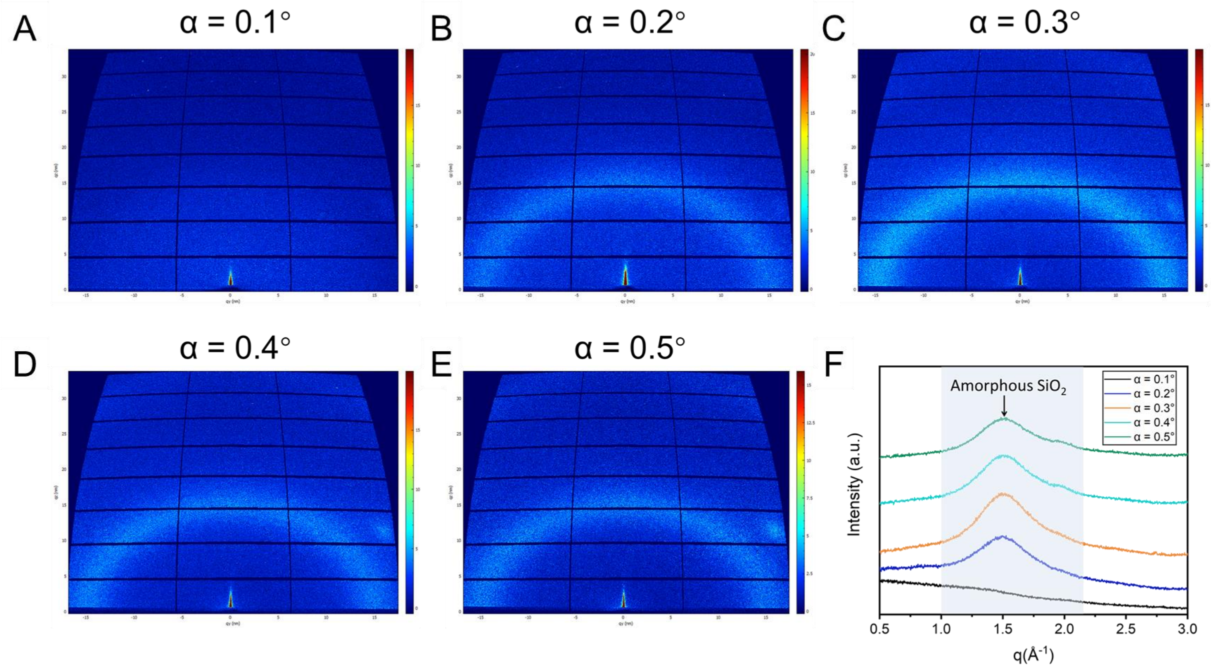

Fig. S17. GIWAXS patterns of the substrate (graphene/SiO_2/Si wafer). (A-E) Incident angle α from 0.1-0.5°, respectively, (F) integrated from panel (A-E).

***Note:** From the outset, we observe that at an incidence angle (α) ranging from 0.02 to 0.06°, the 002 peak manifests as a single entity. As the incidence angle extends beyond 0.08°, the peak begins to bifurcate into two distinct peaks. At an increased incidence angle of 0.14°, the discrepancy between the two 002 peaks becomes more pronounced. Beyond an incidence angle of 0.16°, the peaks once again coalesce into a single entity. Concurrently, as depicted in panel a, beginning at an incidence angle of 0.14°, the emergence of a robust, broad peak corresponding to amorphous SiO_2 is evident. This occurrence correlates with the deeper penetration of the beam as the incidence angle escalates.

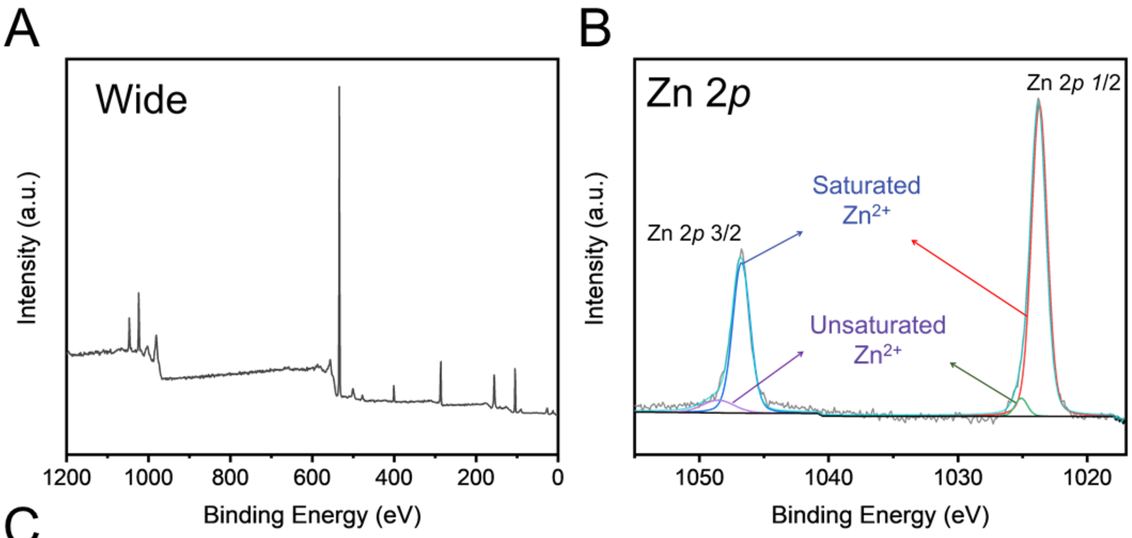

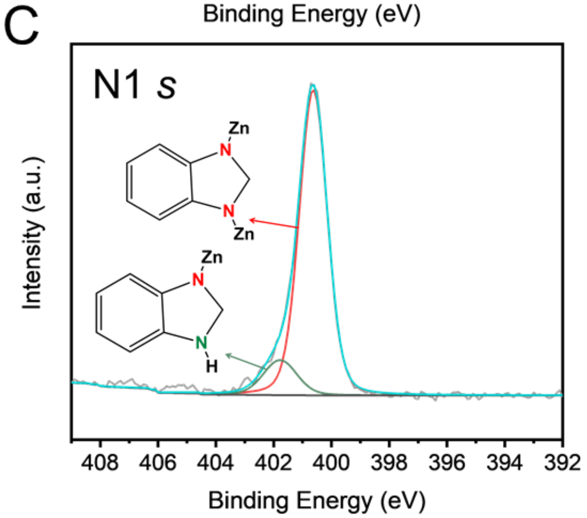

Zinc type	Saturated Zn^{2+} (2*p* 1/2)	Unsaturated Zn^{2+} (2*p* 1/2)	Saturated Zn^{2+} (2*p* 1/2)	Unsaturated Zn^{2+} (2*p* 1/2)
Area	18255.5	2781.4	34073.8	1573.0
Concentration (%)	86.8	13.2	95.6	4.4

Nitrogen type	N-Zn	N-H
Area	7535	1138
Concentration (%)	86.8	13.2

Fig. S18. (A) Wide scan XPS Spectrum of $Zn_2(bim)_4$ film on HOPG. High-resolution XPS spectra of (B) Zn 2*p* and (C) N 1*s*. The table on the right shows the area and the relative concentration of the Zinc and Nitrogen.

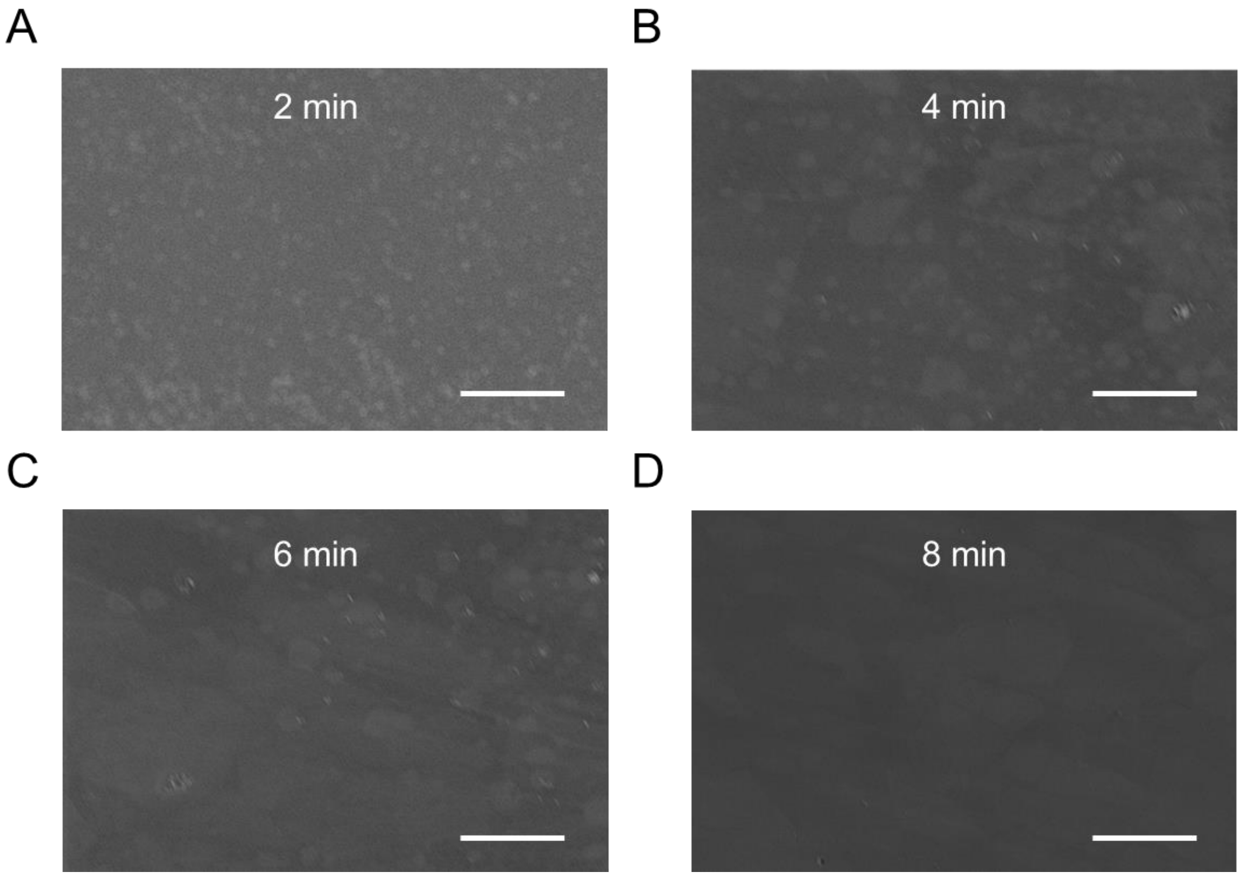

Fig. S19. SEM image of the $Zn_2(bim)_4$/HOPG for different time. (A) 2 minutes, (B) 4 minutes, (C) 6 minutes, (D) 8 minutes. (Scale bar = 2 μm)

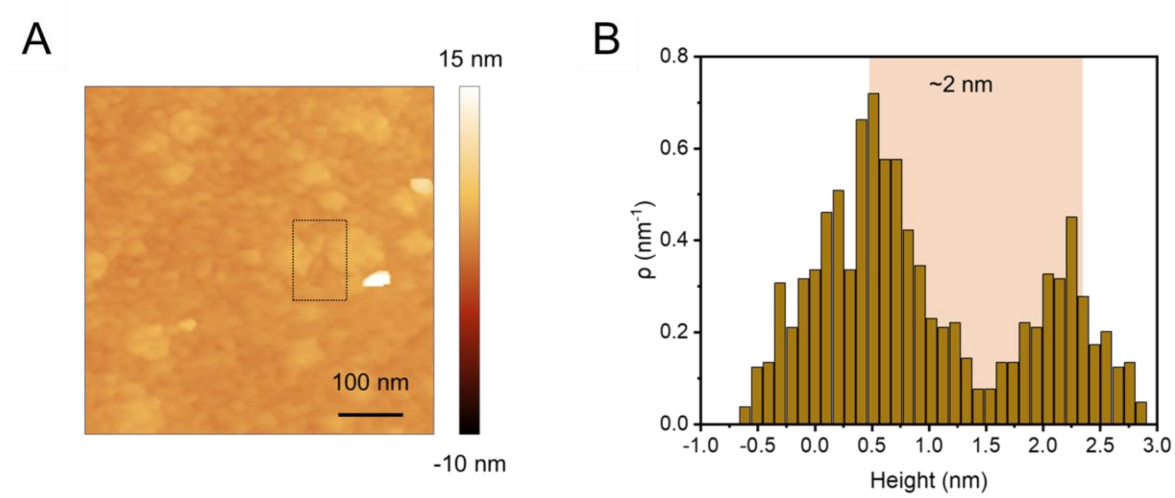

Fig. S20. Morphology of the 2 min growth $Zn_2(bim)_4$ film. (A) AFM image of the $Zn_2(bim)_4$/HOPG and (B) the corresponding height profile of the grains.

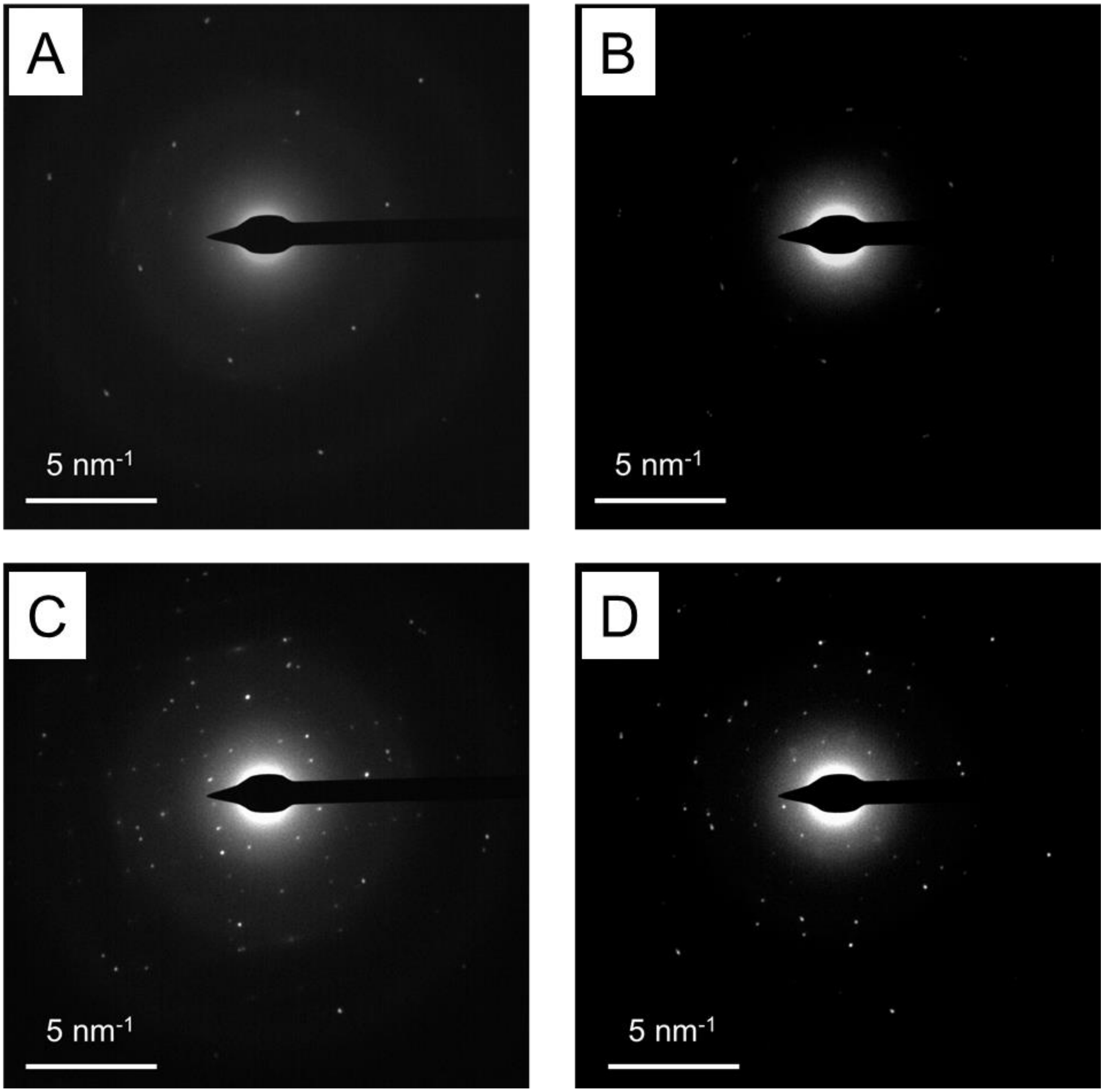

Fig. S21. SAED of the $Zn_2(bim)_4$/graphene for different time. (A) 2 min, (B) 4 min, (C) 6 min and (D) 8 min.

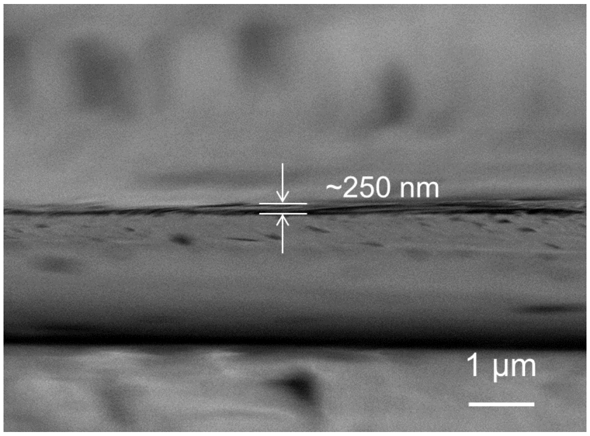

Fig. S22. Cross-sectional SEM image of the NG/PTMSP membrane.

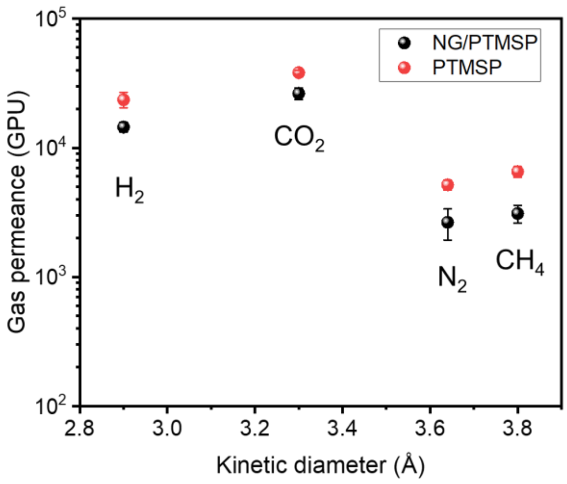

Fig. S23. Comparison of the single gas permeation between standalone polymeric film (PTMSP) and NG/PTMSP film.

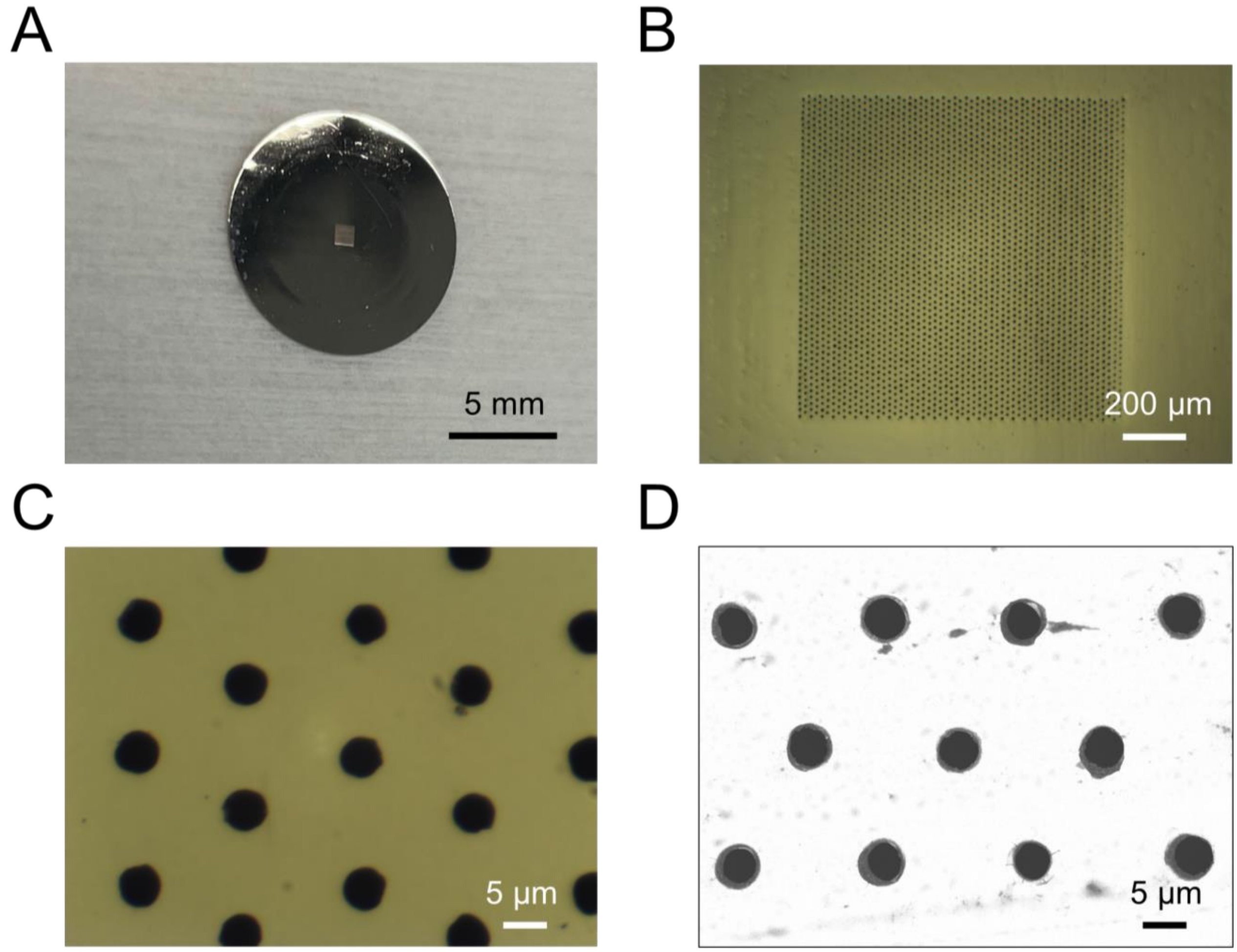

Fig. S24. (A) Photograph**,** (B) and (C) optical microscopy and (D) SEM images of the macroporous W support. The diameter of the pores is 5 μm.

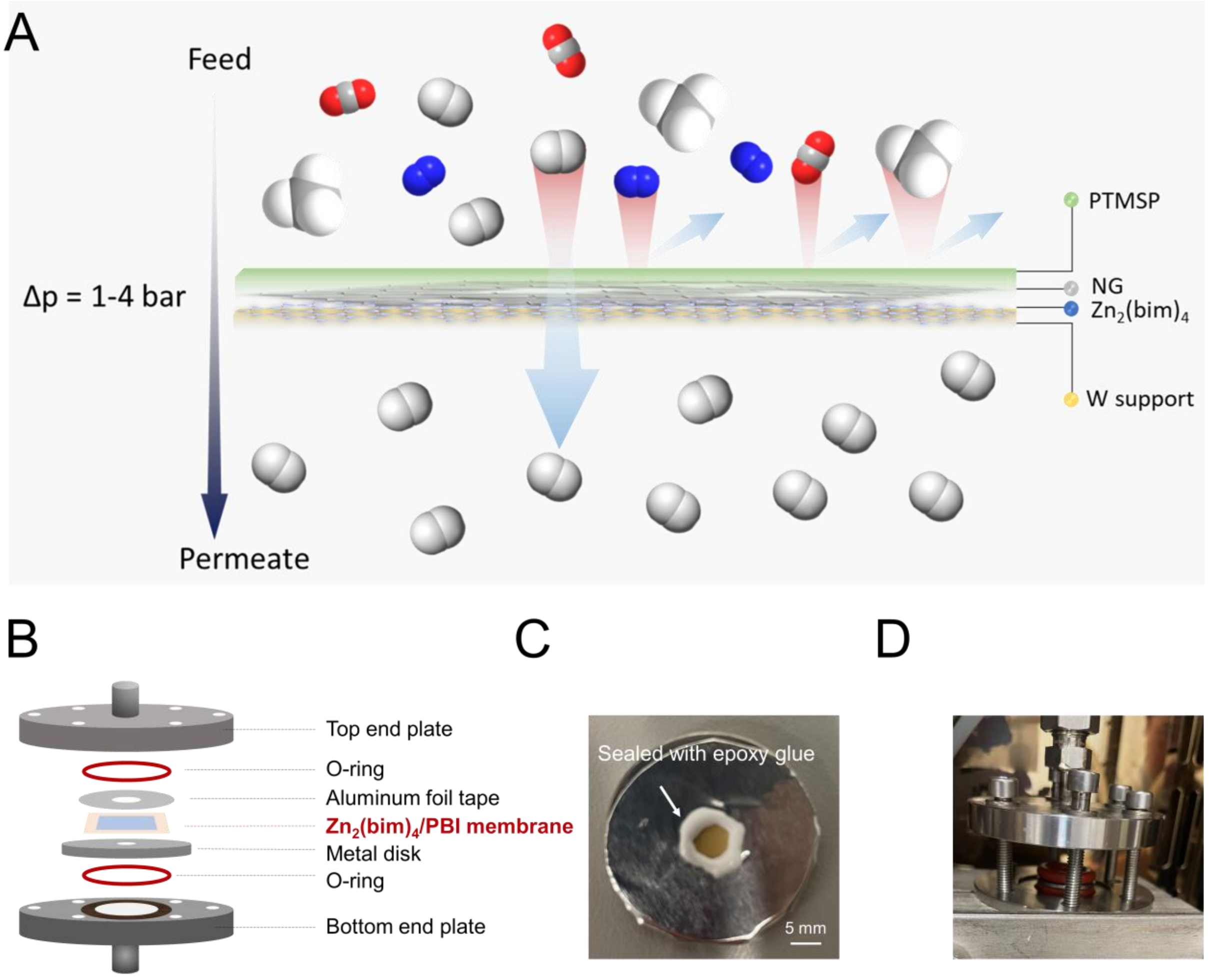

Fig. S25. (A). Schematic of a $Zn_2(bim)_4$ film supported on NG reinforced with PTMSP scooped by W support. (B-D) Description of the membrane module for polymeric supported $Zn_2(bim)_4$ film: (B) Schematic diagram of the membrane module used for the test, (C) configuration of the $Zn_2(bim)_4$/PBI membrane test on the metal disk, the interval between membrane and Al foil is sealed with epoxy glue, (D) picture of the membrane module.

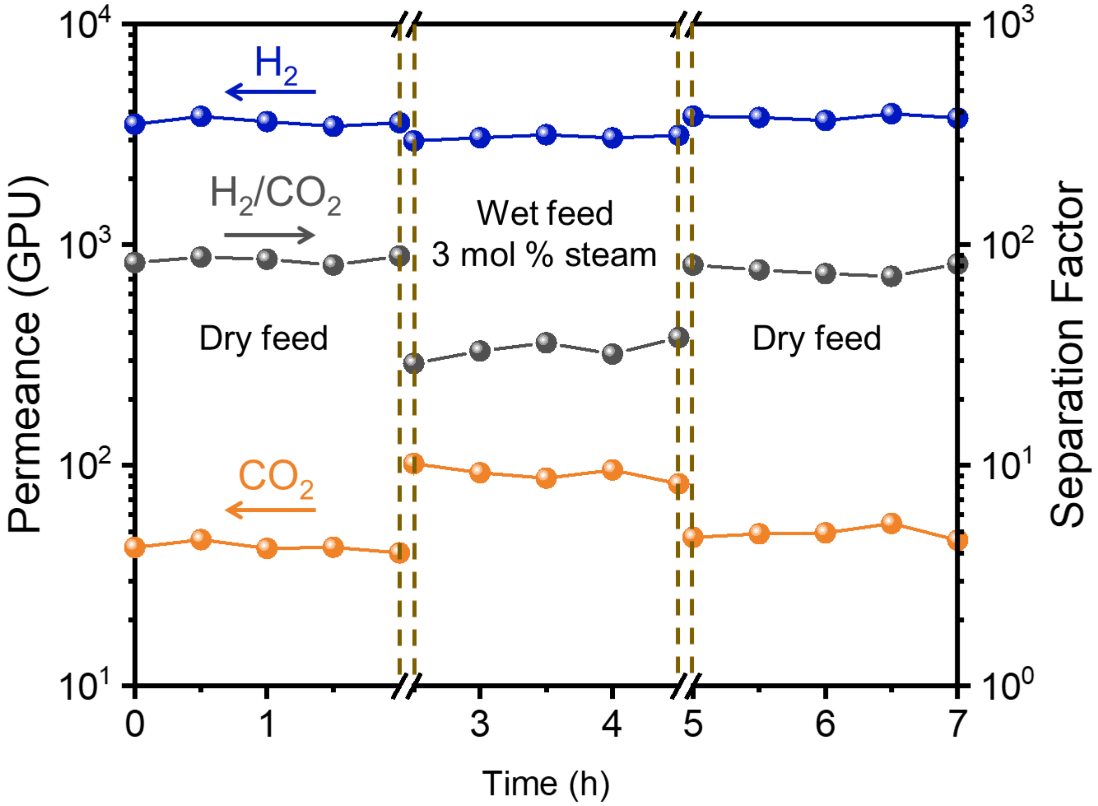

Fig. S26. Equimolar H_2/CO_2 mixture separation tests of a $Zn_2(bim)_4$/NG membrane. (Test conditions: 150°C, ΔP = 1 bar)

***Note:** The H_2/CO_2 separation factor decreased when the humid gas feed was introduced. A slight increase in CO_2 permeance and a decrease in H_2 permeance was observed. Upon returning to dry feed conditions, the separation performance fully recovered.

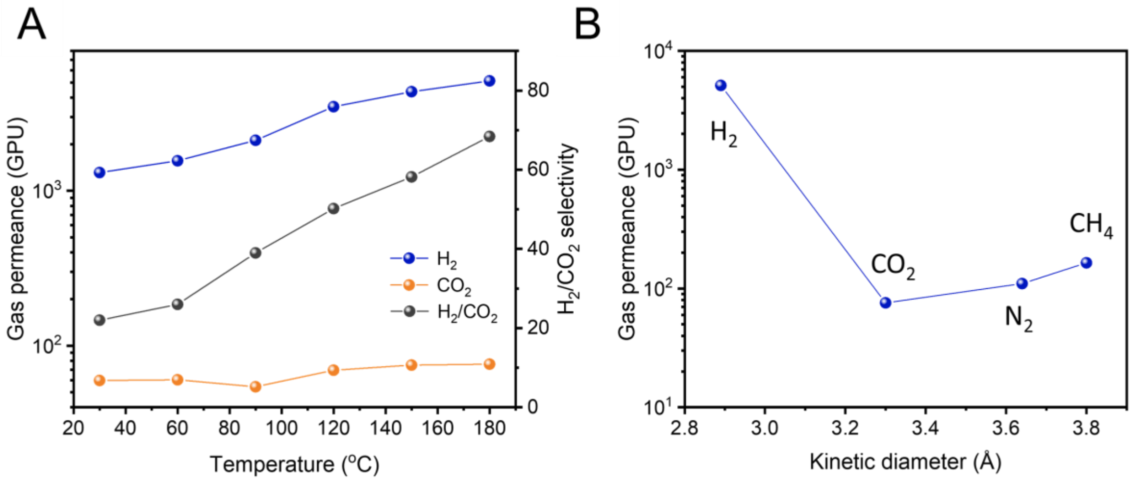

Fig. S27. (A) H_2/CO_2 separation performance of $Zn_2(bim)_4$ sample on polymeric support with the temperature swinging from 25 to 180°C (ΔP of 2 bar). (B) H_2, CO_2, N_2 and CH_4 single gas permeances of the sample at 180°C.

Supplementary Tables

Table S1. The thicknesses of the reported ultra-thin MOF membranes via different deposition methods.

Fabrication method	MOFs	Thickness (nm)	Ref.
Secondary growth	KAUST-7&KAUST-8	800	(*77*)
	ZIF-8	550	(*78*)
	ZIF-95	1000	(*79*)
	MIL-96	180	(*80*)
Interfacial synthesis	ZIF-95	530	(*81*)
	UiO-66	165	(*82*)
	ZIF-8	446	(*83*)
Nanosheet stacking	NH2-MIL-53(Al)	220	(*84*)
	$Zn_2(bim)_3$	10	(*38*)
	MAMS-1	40	(*40*)
	$Zn_2(bim)_4$	10	(*35*)
	ZIF-8	500	(*85*)
	Al-MOF	100	(*86*)
Layer-by-Layer	Cu-TCPP	80	(*87*)
	ZIF-8/(TA-Zn2+)2	85	(*88*)
	ZIF-8/C_3N_4	240	(*89*)
	HKUST-1	500	(*90*)
Vapor deposition	$Co_2(bim)_4$	57	(*91*)
	ZIF-8	100	(*56*)
	ZIF-8	17	(*92*)
Electro-chemical deposition	ZIF-8	360	(*93*)
	ZIF-8	205	(*94*)
	ZIF-8	500	(*95*)
	Zr-fum-fcu-MOF	150	(*55*)
	ZIF-8	180	(*96*)
Direct synthesis	UiO-67	200	(*97*)
	DZIF-8	83	(*70*)
	UiO-66-NH2	500	(*98*)
	UiO-66-SO3H	600	(*99*)
	$Zn_2(bim)_4$	50	(*100*)
	ML-UiO-66	103	(*101*)
	UiO-66	120	(*101*)
	2DZIF	2	(*60*)
This work	$Zn_2(bim)_4$	4	This work
	$Zn_2(bim)_4$	6.6	This work
	$Zn_2(bim)_4$	10,2	This work

Table S2. The calculated lattice parameter based on SAED, GIWAXS and comparison with simulated $Zn_2(bim)_4$.

	$Zn_2(bim)_4$ (on graphene)		$Zn_2(bim)_4$ simulated	Graphene
	ED	GIXRD		
a (Å)	16.182	16.474	16.106	2.46
b (Å)	16.015	16.481	16.126	2.46
c (Å)	-	19.957	19.511	-

Table S3. H_2/CO_2 separation performances of the state-of-the-art membranes. Only literature that used pressurized feed are compared.

Catergory	Membrane	Temperature (°C)	Transmembrane pressure difference(bar)	H_2 permeance (GPU)	Selectivity	References
COF	ppPAF	25	1	709	78.8	(*102*)
	iPAF-6(Br)	25	1	58658	4.1	(*103*)
	CTF-BTD	25	1	655.6	43.1	(*104*)
Polymer	P84-PDMS	35	1	21	5.7	(*105*)
	TB-OR	35	6.89	0.448	5.1	(*106*)
	m-PBI	180	3	48.5	33.3	(*107*)
	PBI/sPPSU	90	14	16.7	9.7	(*108*)
	PBDI	150	1	241	23	(*109*)
	PIM-EA (H_2)-TB/PBI	250	6	57.9	23.8	(*110*)
	MPD-TMC polyamide TFC	140	7.9	350	50	(*111*)
	BILP-101x	150	1	24	40	(*112*)
Zeolite	NaA	25	1	406	7.1	(*113*)
	LTA	30	3	7250	15.3	(*114*)
	ZSM-5-Silicate	450	1	371.2	25.3	(*115*)
MOFs	KAUST-7	25	1	638	17.7	(*77*)
	ZIF-8/GO	25	1	408	30.8	(*116*)
	AO-PIM-1@ZIF-8	25	1	1671	12	(*117*)
	ZIF-95	25	1	571	184	(*79*)
	$Zn_2(bim)_4$/NG	20	1	5590	152	This work (metal porous support)
	$Zn_2(bim)_4$/NG	20	4	3550	62	This work (metal porous support)
	$Zn_2(bim)_4$/NG	180	1	8900	189	This work (metal porous support)
	$Zn_2(bim)_4$/NG	180	1	5220	68	This work (polymeric porous support)

Table S4. Comparison of the H_2 flux and the corresponding H_2/CO_2 selectivity of our membranes with the state-of-the-art membranes in the literature. Only literature that used pressurized feed are compared.

H_2/CO_2 selectivity	H_2 permeance (GPU)	H_2 permeance (mol m^{-2} s^{-1} Pa^{-1})	Transmembrane pressure difference ($Pa \times 10^5$)	H_2 flux (mol m^{-2} s^{-1})	Categories	Mixed/Single gas	Ref.
102	5520	1.85E-06	1	3.70E-01	MOFs	Mixed gas	This work
92	3590	1.20E-06	2	3.61E-01			
78	2850	9.55E-07	3	3.82E-01			
60	2422	8.11E-07	4	4.06E-01			
80	5920	1.98E-06	1	3.97E-01			
146	6290	2.11E-06	1	4.21E-01		Single gas	
59.6	1311	4.39E-07	1	8.78E-02			
60.2	1564	5.24E-07	1	1.05E-01			
54.3	2118	7.10E-07	1	1.42E-01			
69.5	3490	1.17E-06	1	2.34E-01			
75	4367	1.46E-06	1	2.92E-01			
76	5129	1.72E-06	1	3.44E-01			
78.8	709	2.36E-07	1	4.75E-02	COF	Mixed gas	(*102*)
4.1	58658	1.97E-05	1	3.93E+00			(*103*)
43.1	655.6	2.20E-07	1	4.39E-02			(*104*)
5.7	21	7.03E-09	1	1.41E-03	Polymer		(*105*)
5.1	0.448	1.50E-10	6.89	1.18E-04			(*106*)
33.3	48.5	1.62E-08	2	4.87E-03			(*107*)
9.7	16.7	5.59E-09	13	7.83E-03			(*108*)
23	241	8.07E-08	1	1.61E-02			(*109*)
23.8	57.9	1.94E-08	6	1.36E-02			(*110*)
50	350	1.17E-07	7.9	1.04E-01			(*111*)
40	24	8.04E-09	1	1.61E-03			(*112*)
7.1	406	1.36E-07	1	2.72E-02	Zeolite		(*113*)
15.3	7250	2.43E-06	3	9.72E-01			(*114*)
25.3	371.2	1.24E-07	1	2.49E-02			(*115*)
17.7	638	2.14E-07	1	4.27E-02	Other MOFs		(*77*)
30.8	408	1.37E-07	1	2.73E-02			(*116*)
12	1671	5.60E-07	1	1.12E-01			(*117*)
184	571	1.91E-07	1	3.83E-02			(*79*)

Table S5. Single gas permeation data of the parallel samples reported in Fig. 4A.

Membrane No.	H_2 permeance (GPU)		CO_2 permeance (GPU)		N_2 permeance (GPU)		CH_4 permeance (GPU)	
$Zn_2(bim)_4$ - M1	6815		50.4		46.0		67.8	
$Zn_2(bim)_4$ - M2	6625	6290±860	37.6	39±11	65.3	46±19	34.7	45±22
$Zn_2(bim)_4$ - M3	5430		32.3		27.1		34.2	
Support film - M1	15780		29035		3500		3585	
Support film – M2	13620	14500±1280	24520	26350±2685	1930	2650±720	2727	3100±485
Support film – M3	14100		25495		2520		2988	

REFERENCES AND NOTES

1. A. Züttel, A. Remhof, A. Borgschulte, O. Friedrichs, Hydrogen: The future energy carrier. *Philos. Trans. A Math Phys. Eng. Sci.* **368**, 3329–3342 (2010).

2. J. O. Abe, A. Popoola, E. Ajenifuja, O. M. Popoola, Hydrogen energy, economy and storage: Review and recommendation. *Int. J. Hydrogen Energy* **44**, 15072–15086 (2019).

3. J. M. Henis, M. K. Tripodi, The developing technology of gas separating membranes. *Science* **220**, 11–17 (1983).

4. J. A. Rodriguez, S. Ma, P. Liu, J. Hrbek, J. Evans, M. Perez, Activity of CeO_x and TiO_x nanoparticles grown on Au (111) in the water-gas shift reaction. *Science* **318**, 1757–1760 (2007).

5. R. M. Navarro, M. Pena, J. Fierro, Hydrogen production reactions from carbon feedstocks: Fossil fuels and biomass. *Chem. Rev.* **107**, 3952–3991 (2007).

6. K. Liu, C. Song, V. Subramani, *Hydrogen and syngas production and purification technologies* (John Wiley & Sons, 2009).

7. P. Mondal, G. Dang, M. Garg, Syngas production through gasification and cleanup for downstream applications—Recent developments. *Fuel Process. Technol.* **92**, 1395–1410 (2011).

8. H. Nazir, C. Louis, S. Jose, J. Prakash, N. Muthuswamy, M. E. Buan, C. Flox, S. Chavan, X. Shi, P. Kauranen, Is the H_2 economy realizable in the foreseeable future? Part I: H_2 production methods. *Ind. Eng. Chem. Res.* **45**, 13777–13788 (2020).

9. D. J. Couling, K. Prakash, W. H. Green, Analysis of membrane and adsorbent processes for warm syngas cleanup in integrated gasification combined-cycle power with CO_2 capture and sequestration. *Ind. Eng. Chem. Res.* **50**, 11313–11336 (2011).

10. C. Pfeifer, B. Puchner, H. Hofbauer, In-situ CO_2-absorption in a dual fluidized bed biomass steam gasifier to produce a hydrogen rich syngas. *Int. J. Chem. React. Eng.* **5**, (2007).

11. S. D. Kenarsari, D. Yang, G. Jiang, S. Zhang, J. Wang, A. G. Russell, Q. Wei, M. Fan, Review of recent advances in carbon dioxide separation and capture. *RSC Adv.* **3**, 22739–22773 (2013).

12. K. Damen, M. van Troost, A. Faaij, W. Turkenburg, A comparison of electricity and hydrogen production systems with CO_2 capture and storage. Part A: Review and selection of promising conversion and capture technologies. *Prog. Energy Combust. Sci.* **32**, 215–246 (2006).

13. G. Ji, J. G. Yao, P. T. Clough, J. C. D. Da Costa, E. J. Anthony, P. S. Fennell, W. Wang, M. Zhao, Enhanced hydrogen production from thermochemical processes. *Energ. Environ. Sci.* **11**, 2647–2672 (2018).

14. S. Wei, R. Sacchi, A. Tukker, S. Suh, B. Steubing, Future environmental impacts of global hydrogen production. *Energ. Environ. Sci.* **17**, 2157–2172 (2024).

15. F. V. Lima, P. Daoutidis, M. Tsapatsis, J. J. Marano, Modeling and optimization of membrane reactors for carbon capture in integrated gasification combined cycle units. *Ind. Eng. Chem. Res.* **51**, 5480–5489 (2012).

16. A. Basile, J. Tong, P. Millet, Inorganic membrane reactors for hydrogen production: An overview with particular emphasis on dense metallic membrane materials. *Handbook Membra. React.* **1**, 42–148 (2013).

17. F. Gallucci, E. Fernandez, P. Corengia, M. van Sint Annaland, Recent advances on membranes and membrane reactors for hydrogen production. *Chem. Eng. Sci.* **92**, 40–66 (2013).

18. I. S. Metcalfe, B. Ray, C. Dejoie, W. Hu, C. de Leeuwe, C. Dueso, F. R. García-García, C.-M. Mak, E. I. Papaioannou, C. R. Thompson, Overcoming chemical equilibrium limitations using a thermodynamically reversible chemical reactor. *Nat. Chem.* **11**, 638–643 (2019).

19. H. Gao, J. Y. Lin, Y. Li, B. Zhang, Electroless plating synthesis, characterization and permeation properties of Pd–Cu membranes supported on ZrO_2 modified porous stainless steel. *J. Membr. Sci.* **265**, 142–152 (2005).

20. S. Yun, S. T. Oyama, Correlations in palladium membranes for hydrogen separation: A review. *J. Membr. Sci.* **375**, 28–45 (2011).

21. Ø. Hatlevik, S. K. Gade, M. K. Keeling, P. M. Thoen, A. Davidson, J. D. Way, Palladium and palladium alloy membranes for hydrogen separation and production: History, fabrication strategies, and current performance. *Sep. Purif. Technol.* **73**, 59–64 (2010).

22. A. Mejdell, T. Peters, M. Stange, H. Venvik, R. Bredesen, Performance and application of thin Pd-alloy hydrogen separation membranes in different configurations. *J. Taiwan Inst. Chem. Eng.* **40**, 253–259 (2009).

23. A. Mejdell, M. Jøndahl, T. Peters, R. Bredesen, H. Venvik, Effects of CO and CO_2 on hydrogen permeation through a ~3 μm Pd/Ag 23 wt.% membrane employed in a microchannel membrane configuration. *Sep. Purif. Technol.* **68**, 178–184 (2009).

24. H. B. Park, J. Kamcev, L. M. Robeson, M. Elimelech, B. D. Freeman, Maximizing the right stuff: The trade-off between membrane permeability and selectivity. *Science* **356**, eaab0530 (2017).

25. L. Zhu, M. T. Swihart, H. Lin, Unprecedented size-sieving ability in polybenzimidazole doped with polyprotic acids for membrane H_2/CO_2 separation. *Energ. Environ. Sci.* **11**, 94–100 (2018).

26. T. Yang, Y. Xiao, T.-S. Chung, Poly-/metal-benzimidazole nano-composite membranes for hydrogen purification. *Energ. Environ. Sci.* **4**, 4171–4180 (2011).

27. N. Du, H. B. Park, M. M. Dal-Cin, M. D. Guiver, Advances in high permeability polymeric membrane materials for CO_2 separations. *Energ. Environ. Sci.* **5**, 7306–7322 (2012).

28. Y. Zhao, W. W. Ho, CO_2-selective membranes containing sterically hindered amines for CO_2/H_2 separation. *Ind. Eng. Chem. Res.* **52**, 8774–8782 (2013).

29. H. Verweij, Y. Lin, J. Dong, Microporous silica and zeolite membranes for hydrogen purification. *MRS Bull.* **31**, 756–764 (2006).

30. J. Choi, M. Tsapatsis, MCM-22/silica selective flake nanocomposite membranes for hydrogen separations. *J. Am. Chem. Soc.* **132**, 448–449 (2010).

31. M. Dakhchoune, L. F. Villalobos, R. Semino, L. Liu, M. Rezaei, P. Schouwink, C. E. Avalos, P. Baade, V. Wood, Y. Han, M. Ceriotti, K. V. Agrawal, Gas-sieving zeolitic membranes fabricated by condensation of precursor nanosheets. *Nat. Mater.* **20**, 362–369 (2021).

32. X. Duan, M. Dakhchoune, J. Hao, K. V. Agrawal, Scalable room-temperature synthesis of a hydrogen-sieving zeolitic membrane on a polymeric support. *ACS Sustainable Chem. Eng.* **11**, 8140–8147 (2023).

33. X. Tan, S. Robijns, R. Thür, Q. Ke, N. de Witte, A. Lamaire, Y. Li, I. Aslam, D. van Havere, T. Donckels, T. van Assche, V. van Speybroeck, M. Dusselier, I. Vankelecom, Truly combining the advantages of polymeric and zeolite membranes for gas separations. *Science* **378**, 1189–1194 (2022).

34. X. Gu, Z. Tang, J. Dong, On-stream modification of MFI zeolite membranes for enhancing hydrogen separation at high temperature. *Microporous Mesoporous Mater.* **111**, 441–448 (2008).

35. Y. Peng, Y. Li, Y. Ban, H. Jin, W. Jiao, X. Liu, W. Yang, Metal-organic framework nanosheets as building blocks for molecular sieving membranes. *Science* **346**, 1356–1359 (2014).

36. M. Zhao, D.-D. Zhou, P. Chen, Y. Ban, Y. Wang, Z. Hu, Y. Lu, M.-Y. Zhou, X.-M. Chen, W. Yang, Heat-driven molecule gatekeepers in MOF membrane for record-high H_2 selectivity. *Sci. Adv.* **9**, eadg2229 (2023).

37. G. Chen, C. Chen, Y. Guo, Z. Chu, Y. Pan, G. Liu, G. Liu, Y. Han, W. Jin, N. Xu, Solid-solvent processing of ultrathin, highly loaded mixed-matrix membrane for gas separation. *Science* **381**, 1350–1356 (2023).

38. Y. Peng, Y. Li, Y. Ban, W. Yang, Two-dimensional metal–organic framework nanosheets for membrane-based gas separation. *Angew. Chem. Int. Ed. Engl.* **129**, 9889–9893 (2017).

39. Y. S. Li, F. Y. Liang, H. Bux, A. Feldhoff, W. S. Yang, J. Caro, Molecular sieve membrane: Supported metal–organic framework with high hydrogen selectivity. *Angew. Chem. Int. Ed. Engl.* **3**, 558–561 (2010).

40. X. Wang, C. Chi, K. Zhang, Y. Qian, K. M. Gupta, Z. Kang, J. Jiang, D. Zhao, Reversed thermo-switchable molecular sieving membranes composed of two-dimensional metal-organic nanosheets for gas separation. *Nat. Commun.* **8**, 14460 (2017).

41. L. Chen, G. Shi, J. Shen, B. Peng, B. Zhang, Y. Wang, F. Bian, J. Wang, D. Li, Z. Qian, G. Xu, G. Liu, J. Zeng, L. Zhang, Y. Yang, G. Zhou, M. Wu, W. Jin, J. Li, H. Fang, Ion sieving in graphene oxide membranes via cationic control of interlayer spacing. *Nature* **550**, 380–383 (2017).

42. J. Kang, Y. Ko, J. P. Kim, J. Y. Kim, J. Kim, O. Kwon, K. C. Kim, D. W. Kim, Microwave-assisted design of nanoporous graphene membrane for ultrafast and switchable organic solvent nanofiltration. *Nat. Commun.* **14**, 901 (2023).

43. W.-H. Zhang, M.-J. Yin, Q. Zhao, C.-G. Jin, N. Wang, S. Ji, C. L. Ritt, M. Elimelech, Q.-F. An, Graphene oxide membranes with stable porous structure for ultrafast water transport. *Nat. Nanotechnol.* **16**, 337–343 (2021).

44. B. Mi, Graphene oxide membranes for ionic and molecular sieving. *Science* **343**, 740–742 (2014).

45. H. W. Kim, H. W. Yoon, S.-M. Yoon, B. M. Yoo, B. K. Ahn, Y. H. Cho, H. J. Shin, H. Yang, U. Paik, S. Kwon, J.Y. Choi, H. B. Park, Selective gas transport through few-layered graphene and graphene oxide membranes. *Science* **342**, 91–95 (2013).

46. R. Joshi, P. Carbone, F.-C. Wang, V. G. Kravets, Y. Su, I. V. Grigorieva, H. Wu, A. K. Geim, R. R. Nair, Precise and ultrafast molecular sieving through graphene oxide membranes. *Science* **343**, 752–754 (2014).

47. L. Ding, L. Li, Y. Liu, Y. Wu, Z. Lu, J. Deng, Y. Wei, J. Caro, H. Wang, Effective ion sieving with $Ti_3C_2T_x$ MXene membranes for production of drinking water from seawater. *Nat. Sustain.* **3**, 296–302 (2020).

48. Y. Li, H. Shao, Z. Lin, J. Lu, L. Liu, B. Duployer, P. O. Å. Persson, P. Eklund, L. Hultman, M. Li, K. Chen, X.-H. Zha, S. Du, P. Rozier, Z. Chai, E. Raymundo-Piñero, P.-L. Taberna, P. Simon, Q. Huang, A general Lewis acidic etching route for preparing MXenes with enhanced electrochemical performance in non-aqueous electrolyte. *Nat. Mater.* **19**, 894–899 (2020).

49. S. Wan, X. Li, Y. Chen, N. Liu, Y. Du, S. Dou, L. Jiang, Q. Cheng, High-strength scalable MXene films through bridging-induced densification. *Science* **374**, 96–99 (2021).

50. M. A. Khayum, S. Kandambeth, S. Mitra, S. B. Nair, A. Das, S. S. Nagane, R. Mukherjee, R. Banerjee, Chemically delaminated free-standing ultrathin covalent organic nanosheets. *Angew. Chem. Int. Ed.* **55**, 15604–15608 (2016).

51. S. Mitra, S. Kandambeth, B. P. Biswal, A. Khayum M, C. K. Choudhury, M. Mehta, G. Kaur, S. Banerjee, A. Prabhune, S. Verma, S. Roy, U. K. Kharul, R. Banerjee, Self-exfoliated guanidinium-based ionic covalent organic nanosheets (iCONs). *J. Am. Chem. Soc.* **138**, 2823–2828 (2016).

52. P. Wang, Y. Peng, C. Zhu, R. Yao, H. Song, L. Kun, W. Yang, Single-phase covalent organic framework staggered stacking nanosheet membrane for CO_2-selective separation. *Angew. Chem. Int. Ed. Engl.* **60**, 19047–19052 (2021).

53. Z. Wang, X. Yan, Q. Hou, Y. Liu, X. Zeng, Y. Kang, W. Zhao, X. Li, S. Yuan, R. Qiu, Scalable high yield exfoliation for monolayer nanosheets. *Nat. Commun.* **14**, 236 (2023).

54. Q. Qian, P. A. Asinger, M. J. Lee, G. Han, K. Mizrahi Rodriguez, S. Lin, F. M. Benedetti, A. X. Wu, W. S. Chi, Z. P. Smith, MOF-based membranes for gas separations. *Chem. Rev.* **120**, 8161–8266 (2020).

55. S. Zhou, O. Shekhah, J. Jia, J. Czaban-Jóźwiak, P. M. Bhatt, A. Ramírez, J. Gascon, M. Eddaoudi, Electrochemical synthesis of continuous metal-organic framework membranes for separation of hydrocarbons. *Nat. Energy* **6**, 882–891 (2021).

56. X. Ma, P. Kumar, N. Mittal, A. Khlyustova, P. Daoutidis, K. A. Mkhoyan, M. Tsapatsis, Zeolitic imidazolate framework membranes made by ligand-induced permselectivation. *Science* **361**, 1008–1011 (2018).

57. G. Huang, B. Ghalei, A. Pournaghshband Isfahani, H. E. Karahan, D. Terada, D. Qin, C. Li, M. Tsujimoto, D. Yamaguchi, K. Sugimoto, R. Igarashi, B. K. Chang, T. Li, M. Shirakawa, E. Sivaniah, Overcoming humidity-induced swelling of graphene oxide-based hydrogen membranes using charge-compensating nanodiamonds. *Nat. Energy* **6**, 1176–1187 (2021).

58. S. Qiu, M. Xue, G. Zhu, Metal–organic framework membranes: From synthesis to separation application. *Chem. Soc. Rev.* **43**, 6116–6140 (2014).

59. A. Knebel, J. Caro, Metal-organic frameworks and covalent organic frameworks as disruptive membrane materials for energy-efficient gas separation. *Nat. Nanotechnol.* **17**, 911–923 (2022).

60. Q. Liu, Y. Miao, L. F. Villalobos, S. Li, H.-Y. Chi, C. Chen, M. T. Vahdat, S. Song, D. J. Babu, J. Hao, Y. Han, M. Tsapatsis, K. V. Agrawal, Unit-cell-thick zeolitic imidazolate framework films for membrane application. *Nat. Mater.* **22**, 1387–1393 (2023).

61. Q.-F. Yang, X.-B. Cui, J.-H. Yu, J. Lu, X.-Y. Yu, X. Zhang, J.-Q. Xu, Q. Hou, T.-G. Wang, A series of metal–organic complexes constructed from in situ generated organic amines. *CrstEngComm* **10**, 1534–1541 (2008).

62. T. D. Bennett, A. K. Cheetham, A. H. Fuchs, F.-X. Coudert, Interplay between defects, disorder and flexibility in metal-organic frameworks. *Nat. Chem.* **9**, 11–16 (2017).

63. E. O. R. Beake, M. T. Dove, A. E. Phillips, D. A. Keen, M. G. Tucker, A. L. Goodwin, T. D. Bennett, A. K. Cheetham, Flexibility of zeolitic imidazolate framework structures studied by neutron total scattering and the reverse Monte Carlo method. *J. Phys. Condens.Matter* **25**, 395403 (2013).

64. K. Zhang, R. P. Lively, C. Zhang, R. R. Chance, W. J. Koros, D. S. Sholl, S. Nair, Exploring the framework hydrophobicity and flexibility of ZIF-8: From biofuel recovery to hydrocarbon separations. *J. Phys. Chem. Lett.* **4**, 3618–3622 (2013).

65. H. Song, Y. Peng, C. Wang, L. Shu, C. Zhu, Y. Wang, H. He, W. Yang, Structure regulation of MOF nanosheet membrane for accurate H_2/CO_2 separation. *Angew. Chem. Int. Ed. Engl.* **135**, e202218472 (2023).

66. K. S. Park, Z. Ni, A. P. Côté, J. Y. Choi, R. Huang, F. J. Uribe-Romo, H. K. Chae, M. O'Keeffe, O. M. Yaghi, Exceptional chemical and thermal stability of zeolitic imidazolate frameworks. *Proc. Natl. Acad. Sci. U.S.A.* **103**, 10186–10191 (2006).

67. P. Zhao, G. I. Lampronti, G. O. Lloyd, M. T. Wharmby, S. Facq, A. K. Cheetham, S. A. T. Redfern, Phase transitions in zeolitic imidazolate framework 7: The importance of framework flexibility and guest-induced instability. *Chem. Mater.* **26**, 1767–1769 (2014).

68. N. Mrkyvkova, P. Nadazdy, M. Hodas, J. Chai, S. Wang, D. Chi, M. Sojkova, M. Hulman, A. Chumakov, O. V. Konovalov, A. Hinderhofer, M. Jergel, E. Majkova, P. Siffalovic, F. Schreiber, Simultaneous monitoring of molecular thin film morphology and crystal structure by x-ray scattering. *Cryst. Growth Des.* **20**, 5269–5276 (2020).

69. J. A. Steele, E. Solano, D. Hardy, D. Dayton, D. Ladd, K. White, P. Chen, J. Hou, H. Huang, R. A. Saha, L. Wang, F. Gao, J. Hofkens, M. B. J. Roeffaers, D. Chernyshov, M. F. Toney, How to GIWAXS: Grazing incidence wide angle x-ray scattering applied to metal halide perovskite thin films. *Adv. Energy Mater.* **13**, 2300760 (2023).

70. Z. Qiao, Y. Liang, Z. Zhang, D. Mei, Z. Wang, M. D. Guiver, C. Zhong, Ultrathin low-crystallinity MOF membranes fabricated by interface layer polarization induction. *Adv. Mater.* **32**, e2002165 (2020).

71. A. Knebel, A. Bavykina, S. J. Datta, L. Sundermann, L. Garzon-Tovar, Y. Lebedev, S. Durini, R. Ahmad, S. M. Kozlov, G. Shterk, M. Karunakaran, I. D. Carja, D. Simic, I. Weilert, M. Kluppel, U. Giese, L. Cavallo, M. Rueping, M. Eddaoudi, J. Caro, J. Gascon, Solution

processable metal-organic frameworks for mixed matrix membranes using porous liquids. *Nat. Mater.* **19**, 1346–1353 (2020).

72. G. He, S. Huang, L. F. Villalobos, J. Zhao, M. Mensi, E. Oveisi, M. Rezaei, K. V. Agrawal, High-permeance polymer-functionalized single-layer graphene membranes that surpass the postcombustion carbon capture target. *Energ. Environ. Sci.* **12**, 3305–3312 (2019).

73. J. Zhao, G. He, S. Huang, L. Villalobos, M. Dakhchoune, H. Bassas, K. Agrawal, Etching gas-sieving nanopores in single-layer graphene with an angstrom precision for high-performance gas mixture separation. *Sci. Adv.* **5**, eaav1851 (2019).

74. M. Dakhchoune, X. Duan, L. F. Villalobos, C. E. Avalos, K. V. Agrawal, Hydrogen-sieving zeolitic films by coating zeolite nanosheets on porous polymeric support. *J. Membr. Sci.* **672**, 121454 (2023).

75. S. Huang, S. Li, L. F. Villalobos, M. Dakhchoune, M. Micari, D. J. Babu, M. T. Vahdat, M. Mensi, E. Oveisi, K. V. Agrawal, Millisecond lattice gasification for high-density CO_2- and O_2-sieving nanopores in single-layer graphene. *Sci. Adv.* **7**, eabf0116 (2021).

76. V. Dyadkin, P. Pattison, V. Dmitriev, D. Chernyshov, A new multipurpose diffractometer PILATUS@SNBL. *J. Synchrotron Radiat.* **23**, 825–829 (2016).

77. S. Zhou, O. Shekhah, T. Jin, J. Jia, S. J. Datta, P. M. Bhatt, M. Eddaoudi, A CO_2-recognition metal-organic framework membrane for continuous carbon capture. *Chem* **9**, 1182–1194 (2023).

78. D. J. Babu, G. He, J. Hao, M. T. Vahdat, P. A. Schouwink, M. Mensi, K. V. Agrawal, Restricting lattice flexibility in polycrystalline metal–organic framework membranes for carbon capture. *Adv. Mater.* **31**, e1900855 (2019).

79. A. Deng, X. Shen, Z. Wan, Y. Li, S. Pang, X. He, J. Caro, A. Huang, Elimination of grain boundary defects in zeolitic imidazolate framework ZIF-95 membrane via solvent-free secondary growth. *Angew. Chem. Int. Ed. Engl.* **133**, 25667–25671 (2021).

80. S. Chen, Y. Liu, Y. Sun, G. Xu, T. Ji, X. Zhang, F. Wang, Y. Lui, Fabrication of MIL-96 nanosheets and relevant c-oriented ultrathin membrane through solvent optimization. *J. Membr. Sci.* **643**, 120064 (2022).

81. X. Ma, Z. Wan, Y. Li, X. He, J. Caro, A. Huang, Anisotropic gas separation in oriented ZIF-95 membranes prepared by vapor-assisted in-plane epitaxial growth. *Angew. Chem. Int. Ed.* **132**, 21044–21048 (2020).

82. Y. Sun, J. Yan, Y. Gao, T. Ji, S. Chen, C. Wang, P. Lu, Y. Li, Y. Liu, Fabrication of highly oriented ultrathin zirconium metal-organic framework membrane from nanosheets towards unprecedented gas separation. *Angew. Chem. Int. Ed.* **62**, e202216697 (2023).

83. H. Zhang, J. Hou, Y. Hu, P. Wang, R. Ou, L. Jiang, J. Z. Liu, B. D. Freeman, A. J. Hill, H. Wang, Ultrafast selective transport of alkali metal ions in metal organic frameworks with subnanometer pores. *Sci. Adv.* **4**, eaaq0066 (2018).

84. A. Pustovarenko, M. G. Goesten, S. Sachdeva, M. Shan, Z. Amghouz, Y. Belmabkhout, A. Dikhtiarenko, T. Rodenas, D. Keskin, I. K. Voets, B. M. Weckhuysen, M. Eddaoudi, L. C. P. M. de Smet, E. J. R. Sudhölter, F. Kapteijn, B. Seoane, J. Gascon, Nanosheets of nonlayered aluminum metal-organic frameworks through a surfactant-assisted method. *Adv. Mater.* **30**, e1707234 (2018).

85. X. Duan, P. Kaya, H.-Y. Chi, B. Topuz, K. V. Agrawal, Fabrication of ZIF-8 membranes by direct assembly of nanosheets from bottom-up synthesis growth solution. *J. Membr. Sci. Lett.* **3**, 100045 (2023).

86. M. Jian, R. Qiu, Y. Xia, J. Lu, Y. Chen, Q. Gu, R. Liu, C. Hu, J. Qu, H. Wang, X. Zhang, Ultrathin water-stable metal-organic framework membranes for ion separation. *Sci. Adv.* **6**, eaay3998 (2020).

87. Y. Song, Y. Sun, D. du, M. Zhang, Y. Liu, L. Liu, T. Ji, G. He, Y. Liu, Fabrication of c-oriented ultrathin TCPP-derived 2D MOF membrane for precise molecular sieving. *J. Membr. Sci.* **634**, 119393 (2021).

88. Y. Xiao, W. Zhang, Y. Jiao, Y. Xu, H. Lin, Metal-phenolic network as precursor for fabrication of metal-organic framework (MOF) nanofiltration membrane for efficient desalination. *J. Membr. Sci.* **624**, 119101 (2021).

89. J. Hou, Y. Wei, S. Zhou, Y. Wang, H. Wang, Highly efficient H_2/CO_2 separation via an ultrathin metal-organic framework membrane. *Chem. Eng. Sci.* **182**, 180–188 (2018).

90. S. Hurrle, S. Friebe, J. Wohlgemuth, C. Wöll, J. Caro, L. Heinke, Sprayable, large-area metal-organic framework films and membranes of varying thickness. *Chem. A Eur. J.* **23**, 2294–2298 (2017).

91. P. Nian, H. Liu, X. Zhang, Bottom-up fabrication of two-dimensional Co-based zeolitic imidazolate framework tubular membranes consisting of nanosheets by vapor phase transformation of Co-based gel for H_2/CO_2 separation. *J. Membr. Sci.* **573**, 200–209 (2019).

92. W. Li, P. Su, Z. Li, Z. Xu, F. Wang, H. Ou, J. Zhang, G. Zhang, E. Zeng, Ultrathin metal-organic framework membrane production by gel-vapour deposition. *Nat. Commun.* **8**, 406 (2017).

93. G. He, M. Dakhchoune, J. Zhao, S. Huang, K. V. Agrawal, Electrophoretic nuclei assembly for crystallization of high-performance membranes on unmodified supports. *Adv. Funct. Mater.* **28**, 1707427 (2018).

94. S. Zhou, Y. Wei, L. Li, Y. Duan, Q. Hou, L. Zhang, L.-X. Ding, J. Xue, H. Wang, J. Caro, Paralyzed membrane: Current-driven synthesis of a metal-organic framework with sharpened propene/propane separation. *Sci. Adv.* **4**, eaau1393 (2018).

95. R. Wei, H. Y. Chi, X. Li, D. Lu, Y. Wan, C. W. Yang, Z. Lai, Aqueously cathodic deposition of ZIF-8 membranes for superior propylene/propane separation. *Adv. Funct. Mater.* **30**, 1907089 (2020).

96. J. Wang, Y. Wang, Y. Liu, H. Wu, M. Zhao, Y. Ren, Y. Pu, W. Li, S. Wang, S. Song, Ultrathin ZIF-8 membrane through inhibited Ostwald ripening for high-flux C_3H_6/C_3H_8 separation. *Adv. Funct. Mater.* **32**, 2208064 (2022).

97. A. Knebel, L. Sundermann, A. Mohmeyer, I. Strauß, S. Friebe, P. Behrens, J. Caro, Azobenzene guest molecules as light-switchable CO_2 valves in an ultrathin UiO-67 membrane. *Chem. Mater.* **29**, 3111–3117 (2017).

98. T. Xu, M. A. Shehzad, D. Yu, Q. Li, B. Wu, X. Ren, L. Ge, T. Xu, Highly cation permselective metal-organic framework membranes with leaf-like morphology. *ChemSusChem* **12**, 2593–2597 (2019).

99. T. Xu, M. A. Shehzad, X. Wang, B. Wu, L. Ge, T. Xu, Engineering leaf-like UiO-66-SO_3H membranes for selective transport of cations. *Nanomicro Lett.* **12**, 51 (2020).

100. Y. Li, L. Lin, M. Tu, P. Nian, A. J. Howarth, O. K. Farha, J. Qiu, X. Zhang, Growth of ZnO self-converted 2D nanosheet zeolitic imidazolate framework membranes by an ammonia-assisted strategy. *Nano Res.* **11**, 1850–1860 (2018).

101. X. Wang, Q. Lyu, T. Tong, K. Sun, L.-C. Lin, C. Y. Tang, F. Yang, M. D. Guiver, X. Quan, Y. Dong, Robust ultrathin nanoporous MOF membrane with intra-crystalline defects for fast water transport. *Nat. Commun.* **13**, 266 (2022).

102. Y. Ma, L. Liu, H. Lei, Y. Tian, S. Ding, N. Zhang, G. Zhu, Facile synthesis of porphyrin-based PAF membrane for hydrogen purification. *Inorg. Chem. Commun.* **141**, 109526 (2022).

103. R. Zhao, T. Ma, F. Cui, Y. Tian, G. Zhu, Porous aromatic framework with tailored binding sites and pore sizes as a high-performance hemoperfusion adsorbent for bilirubin removal. *Adv. Sci.* **7**, 2001899 (2020).

104. Y. Zhao, P. Liu, Y. Ying, K. Wei, D. Zhao, D. Liu, Heating-driven assembly of covalent organic framework nanosheets for gas separation. *J. Membr. Sci.* **632**, 119326 (2021).

105. G. Li, Z. Si, S. Yang, T. Xue, J. Baeyens, P. Qin, Fast layer-by-layer assembly of PDMS for boosting the gas separation of P84 membranes. *Chem. Eng. Sci.* **253**, 117588 (2022).

106. L. Wu, X. Chen, Z. Zhang, S. Xu, C. Ma, N. Li, Enhanced molecular selectivity and plasticization resistance in ring-opened Tröger's base polymer membranes. *J. Membr. Sci.* **634**, 119399 (2021).

107. J. Sánchez-Laínez, M. Etxeberria-Benavides, O. David, C. Téllez, J. Coronas, Green preparation of thin films of polybenzimidazole on flat and hollow fiber supports: Application to hydrogen separation. *ChemSusChem* **14**, 952–960 (2021).

108. A. Naderi, A. A. Tashvigh, T.-S. Chung, M. Weber, C. Maletzko, Molecular design of double crosslinked sulfonated polyphenylsulfone/polybenzimidazole blend membranes for an efficient hydrogen purification. *J. Membr. Sci.* **563**, 726–733 (2018).

109. M. Shan, X. Liu, X. Wang, Z. Liu, H. Iziyi, S. Ganapathy, J. Gascon, F. Kapteijn, Novel high performance poly (p-phenylene benzobisimidazole)(PBDI) membranes fabricated by interfacial polymerization for H_2 separation. *J. Mater. Chem. A* **7**, 8929–8937 (2019).

110. J. Sánchez-Laínez, B. Zornoza, M. Carta, R. Malpass-Evans, N. B. McKeown, C. Téllez, J. Coronas, Hydrogen separation at high temperature with dense and asymmetric membranes based on PIM-EA(H_2)-TB/PBI blends. *Ind. Eng. Chem. Res.* **57**, 16909–16916 (2018).

111. Z. Ali, Y. Wang, W. Ogieglo, F. Pacheco, H. Vovusha, Y. Han, I. Pinnau, Gas separation and water desalination performance of defect-free interfacially polymerized para-linked polyamide thin-film composite membranes. *J. Membr. Sci.* **618**, 118572 (2021).

112. A. Gao, X. Yan, S. Cong, X. Wang, H. Liu, Z. Wang, X. Liu, Designed channels in thin benzimidazole-linked polymer membranes for hot H_2 purification. *J. Membr. Sci.* **668**, 121293 (2023).

113. X. Xu, Y. Bao, C. Song, W. Yang, J. Liu, L. Lin, Synthesis, characterization and single gas permeation properties of NaA zeolite membrane. *J. Membr. Sci.* **249**, 51–64 (2005).

114. M. Sen, K. Dana, N. Das, Development of LTA zeolite membrane from clay by sonication assisted method at room temperature for H_2-CO_2 and CO_2-CH_4 separation. *Ultrason. Sonochem.* **48**, 299–310 (2018).

115. I. Makertihartha, M. Zunita, Z. Rizki, P. Dharmawijaya, in *Journal of Physics Conference Series* (IOP Publishing, 2017), vol. 877, pp. 012076.

116. X. Wu, H. Zhang, Z. Yin, Y. Yang, Z. Wang, ZIF-8/GO sandwich composite membranes through a precursor conversion strategy for H_2/CO_2 separation. *J. Membr. Sci.* **647**, 120291 (2022).

117. S. Xiong, C. Pan, G. Dai, C. Liu, Z. Tan, C. Chen, S. Yang, X. Ruan, J. Tang, G. Yu, Interfacial co-weaving of AO-PIM-1 and ZIF-8 in composite membranes for enhanced H_2 purification. *J. Membr. Sci.* **645**, 120217 (2022).
